# Long noncoding RNAs implicated in embryonic development in *Ybx1* knockout zebrafish

**DOI:** 10.1002/2211-5463.13057

**Published:** 2021-02-26

**Authors:** Chen Huang, Bo Zhu, Dongliang Leng, Wei Ge, Xiaohua Douglas Zhang

**Affiliations:** ^1^ State Key Laboratory of Quality Research in Chinese Medicine Macau Institute for Applied Research in Medicine and Health Macau University of Science and Technology China; ^2^ Faculty of Health Sciences University of Macau Taipa China

**Keywords:** deep RNA sequencing, long noncoding RNAs, REDOX, YBX1, zebrafish embryo development

## Abstract

Y‐box‐binding protein 1 (Ybx1, YB‐1), also known as Y‐box transcription factor, is involved in a variety of biological processes (BPs) and pathways, including embryogenesis, reproduction and development in vertebrates. Several noncoding RNAs regulate Ybx1 signaling. However, the role of long noncoding RNAs (lncRNAs) in embryogenesis remains incompletely understood. Here, we investigated the possible involvement of lncRNAs in *Ybx1*‐mediated regulation of vertebrate development by performing systematic transcriptome analysis of RNA sequencing data derived from *ybx1* homozygous mutant zebrafish on day 5 (day5_*ybx1^−/−^*) and wild‐type zebrafish on days 5 and 6 (day5_*ybx1^+/+^* and day6_*ybx1^+/+^*). We identified several lncRNAs affected by *ybx1* disruption that may target reduction–oxidation‐related genes, such as *duox* (NADPH oxidase) and *noxo1a* (NADPH oxidase organizer). Knockdown of three selected lncRNAs led to morphological deformation of larvae, implying an involvement of these lncRNAs in zebrafish embryo development. In summary, our study provides new insights into the lncRNA‐mediated mechanisms underlying development in *Ybx1*‐deficient zebrafish larvae.

## Graphical abstract

Ybx1 is an important protein to vertebrate development. An integrate transcriptome analysis based on the Ybx1 knockout zebrafish identifies several lncRNAs correlated to REDOX‐related genes, noxo1a, duox. Knockdown of these lncRNAs leads to larvae morphological deformation, suggesting their importance to zebrafish embryonic development.

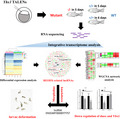

**Abbreviations**
BP: biological process
DE: differentially expressed
*duox*: NADPH oxidase
GO: Gene Ontology
lncRNA: long noncoding RNA
*noxo1a*: NADPH oxidase organizer
nt: nucleotide
PPI: protein–protein interaction
REDOX: reduction–oxidation
ROS/RNS: reactive oxygen and reactive nitrogen species
RNA‐seq: RNA sequencing
TOM: Topology Overlap Matrix
Ybx1: Y‐box‐binding protein 1

Y‐box‐binding protein 1 (Ybx1, YB‐1), also known as Y‐box transcription factor, is a member of a large family of proteins harboring cold shock domain, and it has been proved to have multiple functions involved in a variety of BPs and pathways, including proliferation, differentiation, regulation of apoptosis, translation, stress response, etc. This protein was found in diverse vertebrates and extensively studied in *Homo sapiens* because of its great clinical values. For instance, overexpression of Ybx1 was found in tumor cells and is associated with tumor phenotype [1]. In addition, Ybx1 is demonstrated to harbor the capacity of preventing oncogenic cell transformation via the phosphatidylinositol 3' ‐kinase(PI3K)‐Akt signaling pathway [2]. In as much as the crucial role of Ybx1 is implicated in tumorigenesis, this protein has been regarded as a well‐established biomarker and novel therapeutic target in cancer research [3, 4, 5, 6].

In addition to its medicinal benefits, the multifunctional Ybx1 also has been widely used in the study of vertebrate development because it has been proved to be an essential gene to most vertebrate organisms for growth and development. Typically, many studies showed that *ybx1* knockout in mice could result in severe disorders in embryonic development and even death [7, 8]. On the one hand, Ybx1 was regarded as a crucial protein in early embryogenesis because Ybx1‐deficient mice were found to die at a very early stage of embryogenesis [8]. On the other hand, it was reported that *ybx1^−/−^* embryos exhibit severe growth retardation and progressive mortality after 13.5 embryonic days, which suggests its important role in late‐stage embryonic development in mice [7]. Moreover, maternal Ybx1 has been demonstrated to play a safeguard to protect oocyte maturation and maternal‐to‐zygotic transition in zebrafish [9]. The similar roles of Ybx1 were found in Kumari *et al*.’s study [10]. It was shown that Ybx1 is an essential protein to regulate the maternal control of the Nodal signaling pathway, which has been reported to be essential to the axis formation and germ layer specification in zebrafish [10].

In summary, Ybx1 as a key regulator in embryogenesis and reproduction, as well as development, has already been well demonstrated. However, the underlying mechanism of the processes and pathways mediated by Ybx1 still has much room for deep investigation. For example, Ybx1 exerts multiple functions depending on different subcellular localizations; however, what triggers the translocation of Ybx1 from cytoplasm into nucleus has not been fully understood. Moreover, does noncoding transcriptome participate in Ybx1‐mediated regulation in embryogenesis and development?

Noncoding RNA indeed has emerged in recent years as a crucial regulatory layer to participate in and govern diverse BPs and pathways. In particular, long noncoding RNA (lncRNA) becomes a research hotspot in many fields because it has been demonstrated as a multifunctional regulator involved in a range of BPs and pathways and is associated with diverse diseases [11, 12, 13]. However, regarding whether lncRNA is important to vertebrate development is always debatable, particularly in embryogenesis. Goudarzi *et al*.’s study [14] knocked down 24 specific lncRNAs in zebrafish using CRISPR/Cas9 to investigate their functions in embryo development. The result is not so satisfying because no lncRNAs are found to have overt functions in zebrafish embryogenesis, viability and fertility. Notably, a recent review by Suresh *et al*. [15] demonstrated that a variety of noncoding RNAs mediate Ybx1 signaling, which provides a new perspective to investigate the role of lncRNA in vertebrate development. Hence Ybx1 might be a good breakthrough to uncover the potential lncRNAs and their possible roles in embryonic development. Our study also might be regarded as complementary research providing clues to support the view that some lncRNAs are important to vertebrate development.

In short, our study used Ybx1 as a springboard to investigate the relationship of lncRNAs with zebrafish embryonic development, and successfully uncovered several relevant lncRNAs and their possible roles in it. We believe our study provides new insights into the lncRNA‐mediated mechanisms underlying development in *Ybx1*‐deficient zebrafish larvae.

## Materials and methods

### Animals

The wild‐type zebrafish (*Danio rerio*) AB strain was used in this study. Zebrafish were maintained in the ZebTEC multilinking rack system (Tecniplast, Buguggiate, Italy) under an artificial photoperiod of 14 h light/10 h dark. The temperature, pH and conductivity of the system were 28 ± 1 °C, 7.5 and 400 µS·cm^−1^, respectively. The fish were fed twice a day with Otohime fish diet (Marubeni Nisshin Feed, Tokyo, Japan) by the Tritone automatic feeding system (Tecniplast).

All experiments were performed under a license from the Government of Macau Special Administrative Region and approved by Animal Experimentation Ethics Committee of the University of Macau.

### Larvae RNA preparation and RNA sequencing

Larvae from wild‐type zebrafish of AB strain (day5_*ybx1^+/+^*, day6_*ybx1^+/+^*) and *ybx1* homozygous mutants (day5_*ybx1^−/−^*), which were established by transcription activator‐like effector nucleases, were collected for larval RNA preparation. Around 15–20 zebrafish larvae of each type were collected and pooled for a sufficient amount of RNA per sample. A total of nine samples of day5_*ybx1^+/+^* (three duplicates), day6_*ybx1^+/+^* (three duplicates) and day5_*ybx1^−/−^* (three duplicates) were included in RNA sequencing (RNA‐seq).

Total RNA was extracted using Tri‐Reagent (Molecular Research Center, Cincinnati, OH) according to the protocol of the manufacturer and our previous report [16]. Total RNA was then treated with DNase for 10 min at 37 °C to remove genomic DNA [10 μg RNA in 100 μL reaction buffer with 2 U DNase I from NEB (Ipswich, MA)] followed by phenol‐chloroform extraction and ethanol precipitation.

### Basic RNA‐seq data analysis

Initially, the raw data were processed using Trimmomatic v0.36 (ILLUMINACLIP: TruSeq3‐PE.fa:2:30:10:8:true SLIDINGWINDOW:4:15 LEADING:3 TRAILING:3 MINLEN:50) to remove low‐quality reads and adaptors. Clean reads were aligned against zebrafish genome (Ensembl release 91) [17] using STAR v020201 [18] with two‐pass mode. Then, the alignment results were assembled into transcripts using StringTie v1.3.3b [19] with gene annotation reference. Finally, the assembled transcripts from each sample were merged into a consensus of final transcripts. The quality of the assembled transcripts was evaluated using REF‐EVAL from DETONATE (v1.11) [20].

### lncRNAs identification

To identify lncRNAs, we applied a stringent stepwise filtering pipeline, which has been widely used in our previous studies [21, 22]. At first, the assembled transcripts were annotated and excluded by aligning against known protein sequences from National Center for Biotechnology Information (NCBI) nr database, UniProt database [23], and the mRNA and protein datasets of zebrafish were derived from the Ensembl database using BLAST (v2.6.0+). This step aims at removing protein‐coding sequences as much as possible. Then, the remaining unannotated transcripts with length larger than 200 nucleotides (nt) and longest ORF less than 100 residues were retained. In addition, Coding Potential Calculator [24] was used in the second trimming of protein‐coding sequences. Finally, the remaining transcripts were translated (stop‐to‐stop codon) using an in‐house perl script and then subjected to the Pfam database [25] to search for sequences probably encoding protein domains or motifs. The unmatched transcripts were retained as the final high‐confidence dataset of lncRNAs.

### Differential expression analysis

The assembled transcripts in each sample were quantified by featurecounts (v1.5.3) [26]. Then, the expression profile was normalized using a median of ratios method by r package deseq2 [27]. Differential expression analysis was also performed using deseq2. Differentially expressed (DE) RNAs were obtained by cutoff of adjusted *P* < 0.05 (Benjamin–Hochberg correction) [27].

### Functional prediction of lncRNAs based on network analysis

To investigate the possible functions of lncRNAs implicated in Ybx1 gene knockout, we performed a weighted coexpression network analysis based on the DE lncRNAs and mRNAs using a package wgcna in r [28]. Before wgcna analysis, the expression profiles of lncRNAs and mRNAs were normalized using regularized log normalization in DESeq2. To construct the network and detect the modules related to traits (day5_*ybx1^−/−^* and day6_*ybx1^+/+^*), we used the function *softConnectivity* from wgcna with the ‘randomly selected genes’ parameter set at 5000 and the power parameter precalculated by pickSoft‐Threshold function of wgcna. Then, the predicted modules of highly correlated RNAs were subjected to Metascape to search for relevant BPs and pathways. The intramodular connectivity of mRNA and lncRNA in trait‐related modules was assessed using TOM (Topology Overlap Matrix) similarity, which was calculated by *TOMsimilarityFromExpr* of wgcna. TOM similarity reflects the potential interactions between transcripts.

### Prediction of lncRNA–protein interactions

The lncRNA–protein interactions were predicted using the *cat*RAPID server (*cat*RAPID signature: identification of ribonucleoproteins and RNA‐binding regions). *cat*RAPID (http://s.tartaglialab.com/page/catrapid_group) is an algorithm to estimate the binding propensity of protein–RNA pairs, which has been demonstrated as a program to predict protein–RNA associations with great accuracy.

### Reverse transcription and real‐time quantitative PCR

Reverse transcription was performed at 37 °C for 2 h in a total volume of 10 μL reaction solution containing 3 μg RNA, 0.5 μg oligo (dT), 1X moloney murine leukemia virus reverse trancscriptase (MMLV RT) buffer, 0.5 mm each dNTP, 0.1 mm dithiothreitol and 100 U MMLV RT (Invitrogen, Carlsbad, CA, USA). To validate the RNA‐seq data, we determined the expression levels of several selected lncRNA transcripts by real‐time quantitative PCR in both day5_*ybx1^+/+^* and day5_*ybx1^−/−^*, including MSTRG12630.1, MSTRG24792.1, ENSDART00000171757, MSTRG.30533.1 and MSTRG.33365.1. To study the correlation between the selected lncRNA and their targets, including *duox* (NADPH oxidase), *noxo1a* (NADPH oxidase organizer) and *ybx1*, we determined the mRNA expression levels of targets by real‐time quantitative PCR after targeted lncRNA morpholino microinjection. The expression levels were normalized to that of the housekeeping gene *ef1a*. The standard for each gene was prepared by PCR amplification of cDNA fragments with specific primers (Table 1). The real‐time quantitative PCR assay was performed on the CFX96 Real‐time PCR Detection System (Bio‐Rad, Hercules, CA, USA) and repeated twice. All values were expressed as the mean ± standard error of the mean (*n* = 3), and the data were analyzed by *t*‐test using Prism 6 on Macintosh OS X (GraphPad Software, San Diego, CA, USA).

**Table 1:** Sequence information of primers for real‐time quantitative PCR.

Gene	Primer ID	Strand	Sequence (5'–3')	Size (bp)
MSTRG.12630.1	4975	Sense	TAATATAGTCGCGCGCACAG	94
MSTRG.12630.1	4976	Antisense	TAGAGTCCCCCGTTGTTTTG	94
MSTRG.24792.1	4977	Sense	CAGCAACAAGTCACACTCAACA	143
MSTRG.24792.1	4978	Antisense	CTCTAAACGAGCAAGGTCCAA	143
ENSDART00000171757	4979	Sense	ACGAGGTGTCCTCTCACCAG	134
ENSDART00000171757	4980	Antisense	CCAGGATGGTCCTTGATAGC	134
MSTRG.30533.1	4981	Sense	ACCAAATCAAGCAAGCGTTC	112
MSTRG.30533.1	4982	Antisense	TGTAGGACCTCCGCTTTCAC	112
MSTRG.33365.1	5085	Sense	CCTCTGTTGAACCGGAAAAG	107
MSTRG.33365.1	5086	Antisense	TTTGGAGTTACCTGAAAGGATG	107
duox	4963	Sense	CATGGATGGGGTTTATCTGG	109
duox	4964	Antisense	TCTGTTCTTGTGGGACAGCA	109
noxo1a	5573	Sense	ACACGCAAAAACATGCAGAG	131
noxo1a	5574	Antisense	CCGTCTGTCCATTAGCCATT	131
ybx1	1919	Sense	TCTGATTGCCTTTGTTTTCTGTC	110
ybx1	1920	Antisense	CCCTTCTACCACGTCGAACT	110

### Morpholino knockdown and morphological analysis

To investigate the functions of three selected lncRNAs, ENSDART00000171757, MSTRG.30533.1 and MSTRG.33365.1, we synthesized 300 nmol morpholino and 100 nmol standard morpholino oligo by Gene Tools (Gene Tools, Philomath, OR, USA). The morpholino targeted sequences for synthesis regarding the earlier lncRNA were represented in Table 2. For morpholino microinjection, three different doses (5, 10 and 20 ng) of morpholino were injected into one cell stage embryos for targeted lncRNA, respectively, within 30 min just after spawning. Equal amounts (5, 10 and 20 ng) of standard morpholino oligo were injected as control. On day 5, the embryo number was counted both with normal and deformed morphology individually for different morpholinos and different dose injections. The morphology of morpholino‐injected larvae were photographed by Nikon SMZ18 microscope (Nikon, Tokyo, Japan).

**Table 2:** Morpholino information for three lncRNAs.

Oligo name	Quantity	Production number	Sequence (5'–3')
MSTRG.30533.1	300 nmol	04–13Aug18A	GCTTGATTTGGTTGATTCCTAACTG
MSTRG.33365.1	300 nmol	05–13Aug18A	TGGAGTCCACTGACACTGACTGATC
ENSDART00000171757	300 nmol	06–13Aug18A	ATACTGCATTCCAAACCTGAATCAG

### Immunoblotting

To further study the correlation between lncRNA ENSDART00000171757 and *ybx1* expression at protein level, we used western blotting for evaluation of Ybx1 protein expression between morpholino‐injected (ENSDART00000171757) and control morpholino‐injected groups. The larvae on day 5 with normal morphology were collected for protein sample preparation. The Ybx1 antibodies are from GenScript in Nanjing, China.

In brief, larvae were lysed with 100 µL 1× SDS sample buffer (62.5 mm Tris–HCl, 1% w/v SDS, 10% glycerol, 5% mercaptoethanol, pH 6.8), and the lysates were heated at 95 °C for 10 min and then centrifuged 17 000 ***g*** for 15 min at 4 °C, followed by protein concentration measurement. The samples (total 100 µg proteins) from ENSDART00000171757_morpholino (MO) and control MO were separated on 12% polyacrylamide gels and transferred to poly(vinylidene difluoride) membranes. The membranes were blocked with 5% nonfat milk in 1× TBST for 1 h at room temperature. After washing three times with 1× TBST, the membranes were incubated with Ybx1 primary antibody (1 : 5000) and beta‐actin primary antibody (1 : 2000) in 5 mL of 1× TBST with 5% nonfat milk overnight at 4 °C. They were washed with 1× TBST three times, followed by incubation with the horseradish peroxidase‐conjugated secondary antibody (1 : 2000) in 5 mL 1× TBST for 1 h at room temperature. After washing, the membranes were incubated with the SuperSignal West Femto Maximum Sensitivity Substrate (Thermo Scientific, Waltham, MA, USA), and signals were detected on the ChemiDoc MP imaging system (Bio‐Rad, Hercules, CA, USA).

### Whole‐mount *in situ* hybridization

To further study the spatial expression pattern for the three target lncRNAs, ENSDART00000171757, MSTRG.30533.1 and MSTRG.33365.1, whole‐mount RNA *in situ* hybridization was undertaken for investigation. All their lncRNA probes were transcribed *in vitro* and for storage at −80 °C. Embryos were under 0.003% 1‐phenyl‐2‐thiourea treatment to remove pigment until the larvae reached day 5. On day 5, the *ybx1^+/+^* and *ybx1^−/−^* larvae were fixed in fresh 4% PFA overnight at 4 °C. The larvae were washed with PBST for 5 min at room temperature, and this was repeated four times. For permeabilization, the larvae were immersed with 10 µg·mL^−1^ Protease K in PBST for 15 min at room temperature. The larvae were refixed in 4% PFA for 20 min at room temperature, followed by PBST rinsing for 5 min that was repeated four times. Before mRNA probe hybridization, larvae were immersed in hybridization mix for 2 h at 65 °C. We diluted 100 ng labeled RNA probe in hybridization mix. We removed prehybridization solution and added prewarmed hybridization mix plus probe to the larvae. The hybridization was kept for 40 h at 65 °C. For posthybridization washes, we washed for 5 min in 66% hybridization mix, 33% 2× sodium chloride/sodium citrate (SSC) at 65 °C; washed 5 min in 33% hybridization mix, 66% 2× SSC at 65 °C; and washed 5 min in 2× SSC at 65 °C. We washed 1× 20 min in 0.2× SSC + 0.1% Tween 20 at 65 °C and then washed 2× 20 min in 0.1× SSC + 0.1% Tween 20 at 65 °C. At room temperature, we washed for 5 min in 66% 0.2× SSC, 33% PBST; washed for 5 min in 33% 0.2× SSC, 66% PBST; and washed for 5 min in PBST. After washing, we incubated larvae in blocking solution (PBST plus 2% sheep serum, 2 mg·mL^−1^ BSA) for 1 h at room temperature and prepared the first antibody (alkaline‐phosphatase‐conjugated anti‐digoxigenin) by diluting it in blocking solution, 1 : 5000. We incubated in the antibody for shaking overnight at 4 °C and washed 5 × 15 min in PBST. For colorization, we washed 4 × 5 min in coloration buffer. We mixed 200 µL Nitro Blue tetrazolium/5‐bromo‐4‐chloro‐3‐indolyl phosphate stock mixture with 10 mL coloration buffer. We added 500 µL of this mix to larvae and incubated in the dark at room temperature until a blue reaction product was visible. We stopped the reaction by quickly washing larvae three times by stop solution (PBST, pH 5.5), then washed twice in stop solution for 15 min. After glycerol mounting, we photographed immediately by Nikon SMZ18 microscope (Nikon, Tokyo, Japan).

## Results

### Identification and characterization of lncRNAs

To detect lncRNAs in zebrafish larvae, we conducted a basic bioinformatics analysis. In brief, the RNA‐seq yielded an average of 51 879 984 raw pair‐end reads with length of 150 nt per sample (Table S1). After trimming adaptor sequences and low‐quality reads, the remaining average of 49 379 818 clean reads per sample was aligned to zebrafish genome using STAR and assembled into 77 252 transcripts using Stringtie (Table S2). Then all the assembled transcripts were subjected to quality assessment using DETONATE. The results showed that the majority of transcripts have high quality (Table S3). In addition, the BLAST search against known zebrafish transcripts in the Ensembl database indicated that 61 926 transcripts (~80.16%) have been well recorded in the Ensembl database. Notably, the remaining 16 316 transcripts for which no hits were found in the Ensembl database were probably novel transcripts representing a new subset of functional mRNAs or lncRNAs.

**Fig. 1:**
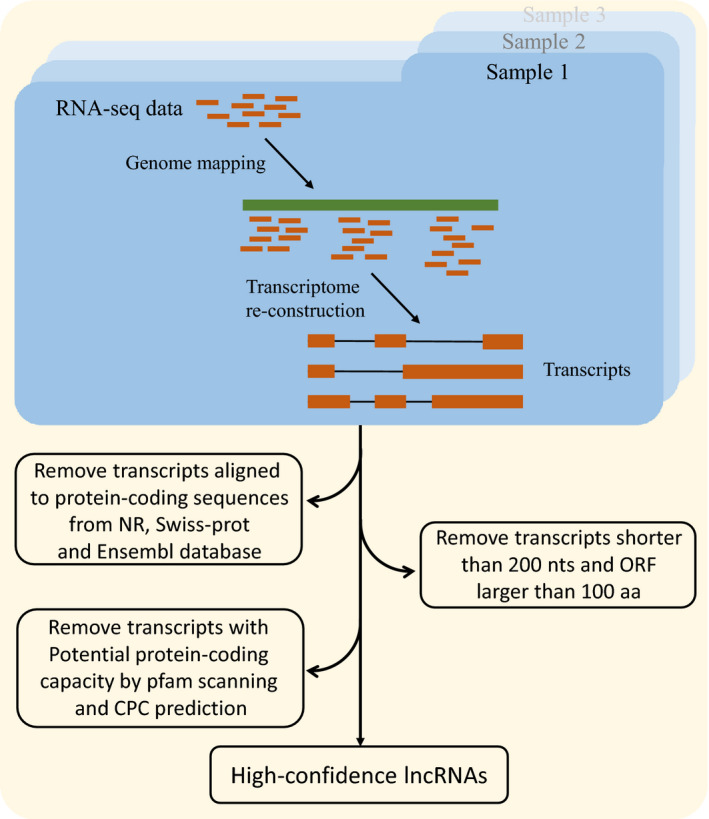
The bioinformatics pipeline used for identification of zebrafish lncRNAs in this study. aa, amino acids; CRC, Coding Potential Calculator.

Based on the well‐established zebrafish transcriptome, a stringent stepwise filtering pipeline was used to identify a high‐confidence lncRNA dataset in this study (Fig. 1). Two core filtering criteria were applied to screen these lncRNAs according to the general definition of lncRNA: (a) the capability of protein coding, and (b) the length of transcripts. In brief, 73 018 potential protein‐coding transcripts obtained by BLAST search against nr, Swiss‐Prot database, as well as the known zebrafish mRNAs/proteins from the Ensembl database, were excluded. The remaining 1555 unannotated transcripts shorter than 200 nt in length and larger than 100 residues in the longest ORF were filtered out. A second protein‐coding filtering was performed to further remove the transcripts with potential protein‐coding capacity, including those containing functional domains/motifs predicted by Pfam scanning and those with protein‐coding potential classified by Coding Potential Calculator. This step filtered out only one transcript. A total of 2678 lncRNAs were finally identified for further study.

We compared those identified lncRNAs with known zebrafish lncRNAs derived from the NONCODE database (v5.0) and ZFLNC database, using BLASTn (e‐value was set to 0.1). The results indicate that a total of 1083 lncRNAs (~40.44%) identified in this study could be well compared with known zebrafish lncRNAs, 460 lncRNAs of which have been included in the NONCODE and ZFLNC database. The remaining mapped lncRNAs exhibit high similarity to the known zebrafish lncRNAs in some specific regions. It is not surprising because lncRNAs have been proved to be quite distinct from mRNAs as mRNAs have a more conserved mutational rate. Instead, lncRNAs have less evolutionary constraints, except the selective pressure to strictly conserve the short functional region, e.g., sequence‐specific interactions, protein‐binding sites, etc. The rest of the lncRNAs that could not be found in any hit in NONCODE and ZFLNC databases might represent a group of novel zebrafish lncRNAs.

### The expression pattern of the DE lncRNAs implicated in Ybx1 gene knockout

**Fig. 2:**
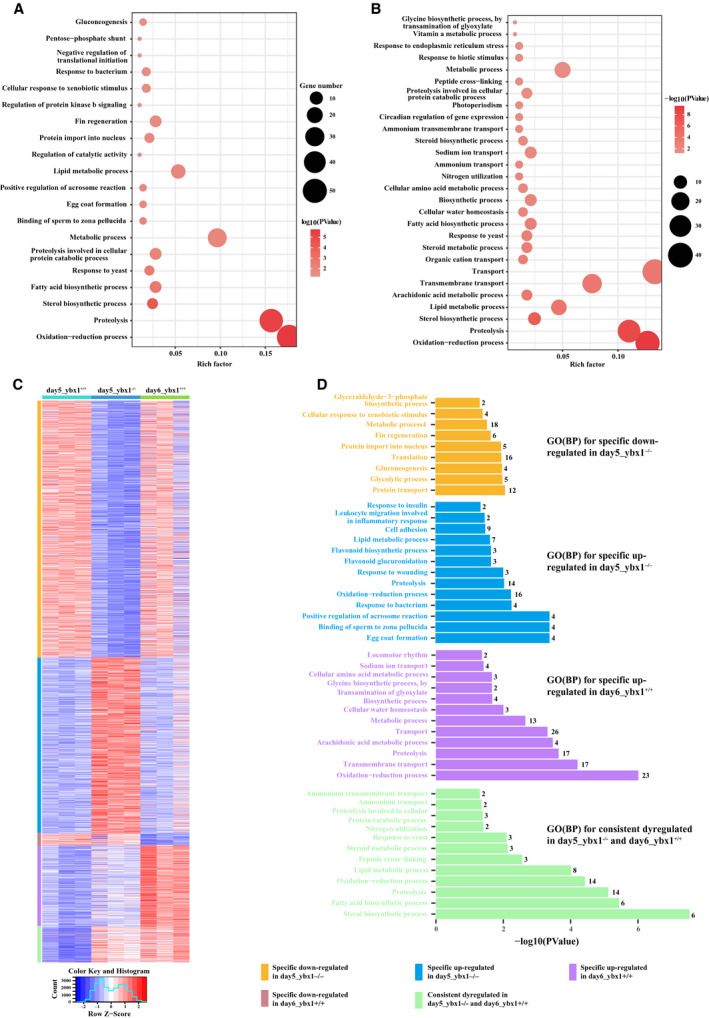
Differential expression analysis between day5_*ybx1* ^+/+^ and day5_*ybx1* *^−/−^* and day6_*ybx1* ^+/+^. GO functional enrichment analysis of DE transcripts achieved on David server: (A) day5_*ybx1* ^+/+^ versus day5_*ybx1* *^−/−^* and (B) day5_*ybx1* ^+/+^ versus day6_*ybx1* ^+/+^. The results are summarized only in the category of BP. The *y* axis indicates functional groups. The *x* axis indicates –log_10_ (*P*‐value). (C) Heatmap of all DE transcripts that were clustered into five subsets, including specific down‐regulated in day5_*ybx1* *^−/−^*, specific up‐regulated in day5_*ybx1* *^−/−^*, specific down‐regulated in day6_*ybx1* ^+/+^, specific up‐regulated in day6_*ybx1* ^+/+^ and consistently dysregulated in day5_*ybx1* *^−/−^* and day6_*ybx1* ^+/+^. The expression profile was normalized using DESeq2 and then hierarchically clustered using heatmap2. Values have been centered and scaled for each row. Each row represents a single transcript. (D) Functional analysis of subsets using DAVID (FDR < 5%).

The expression profile for each sample was initially assessed using featurecounts. To identify the lncRNAs associated with Ybx1 gene knockout, differential expression analysis between day5_*ybx1^+/+^* and day5_*ybx1^−/−^* was conducted using DESeq2. The result shows that 44 lncRNAs (22 are up‐regulated and 22 are down‐regulated) and 1901 mRNAs (849 are up‐regulated and 1052 are down‐regulated) are detected as significantly DE (Figs S1 and S2). Despite no change of the RNA expression level of Ybx1 as observed in day6_*ybx1^+/+^* compared with that of day5_*ybx1^+/+^*, we found that the protein expression level of Ybx1 was dramatically decreased in day6_*ybx1^+/+^* versus day5_*ybx1^+/+^* (data not shown). Hence we also compared the expression level of transcripts between day5_*ybx1^+/+^* and day6_*ybx1^+/+^* to explore the underlying mechanisms resulting in decline of Ybx1 in day6_*ybx1^+/+^* at protein level. DE analysis showed that a total of 542 RNAs were DE in day5_*ybx1^+/+^* versus day6_*ybx1^+/+^*, including 2 DE lncRNAs and 540 DE mRNAs (Figs S3 and S4). Then both results of DE analysis were subjected to the DAVID server to search for the possible functions of those DE RNAs. Intriguingly, both functional results indicated that the hormone synthesis‐related and oxidative stress‐related processes were commonly enriched with an extremely high level of statistical significance, including steroid biosynthesis process, oxidation‐reduction process, etc. (Fig. 2A,B). This result disclosed a close association of Ybx1 with the processes of hormone synthesis and oxidative stress response in zebrafish.

Also, the difference of RNA changes and the corresponding roles caused by two distinct mechanisms resulting in down‐regulation of Ybx1 is another focus of attention for us to investigate. Therefore, we conducted a systematic comparison of day5_*ybx1^+/+^* versus day5_*ybx1^−/−^* and day5_*ybx1^+/+^* versus day6_*ybx1^+/+^* (Fig. 2C). Concretely, 143 transcripts were found to be consistently up/down‐regulated in both comparisons. Remarkably, most of them are up‐regulated, and only five transcripts were observed as down‐regulated. Gene Ontology (GO) functional enrichment analysis for those 143 DE transcripts showed that many BP categories involved in hormone‐related and oxidative stress‐related processes were significantly enriched, such as steroid biosynthesis, oxidation‐reduction process, etc. (Fig. 2D). Furthermore, we found that 731 transcripts (including 22 lncRNAs) were specifically up‐regulated in day5_*ybx1^+/+^* versus day5_*ybx1^−/−^* but exhibit no changes in day5_*ybx1^+/+^* versus day6_*ybx1^+/+^*. Subsequent functional analysis on DAVID indicated that the functions of these specific up‐regulated RNAs involved reproduction processes, that is, egg coat formation, binding of sperm to zona pellucida, etc. Notably, the oxidation–reduction process was also significantly enriched (Fig. 2D). A total of 1062 transcripts (including 22 lncRNAs) were found to be specifically down‐regulated in day5_*ybx1^+/+^* versus day5_*ybx1^−/−^*, which involve protein transport, glycolytic process, gluconeogenesis, etc. (Fig. 2D). Similarly, we have also detected many transcripts DE in day5_*ybx1^+/+^* versus day6_*ybx1^+/+^*, but not present in day5_*ybx1^+/+^* versus day5_*ybx1^−/−^* (Table 3). Concretely, 342 transcripts (including two lncRNAs) exhibit up‐regulation in day6_*ybx1^+/+^*, and functional analysis showed that the functions of those DE RNAs might involve the oxidation–reduction process, transmembrane transport, proteolysis, etc. (Fig. 2D). Forty‐eight specific down‐regulated RNAs were found in day6_*ybx1^+/+^* compared with day5_*ybx1^+/+^*, and their corresponding functional analysis showed that no BP categories were significantly enriched. These functional analyses based on the different subsets of DE transcripts indicated that reduced expression of *y* *bx1* could trigger up‐regulation of oxidative stress‐related BPs and pathways (e.g., the oxidation–reduction process was significantly enriched by specific up‐regulated transcripts in day5_*ybx1^−/−^* and day6_*ybx1^+/+^*). These findings suggest that reduced expression of *y* *bx1*, either caused by Ybx1 knockout or by innate autoregulation, exhibits strong correlation with the reduction–oxidation (REDOX) regulation in zebrafish. Notably, many DE lncRNAs are included in those DE RNA datasets (the majority of them are found in the comparison of day5_*ybx1^+/+^* versus day5_*ybx1^−/−^*), implying specific lncRNAs are present in REDOX regulation after Ybx1 gene knockout.

**Table 3:** Basic statistics of DE transcripts in two different comparisons. 5D WT, Wide Type in 5 Days; 5D MT, Mutant Type in 5 Days; 6D WT, Wide Type in 6 Days.

5D WT versus 5D MT	5D WT versus 6D WT	Number (mRNAs/lncRNAs)
Up	Up	138/0
Down	Down	5/0
Down	Up	7/0
Up	Down	2/0
Up	Non	709/22
Down	Non	1040/22
Non	Up	340/2
Non	Down	48/0

### Network analysis reveals lncRNAs implicated in REDOX regulation

**Fig. 3:**
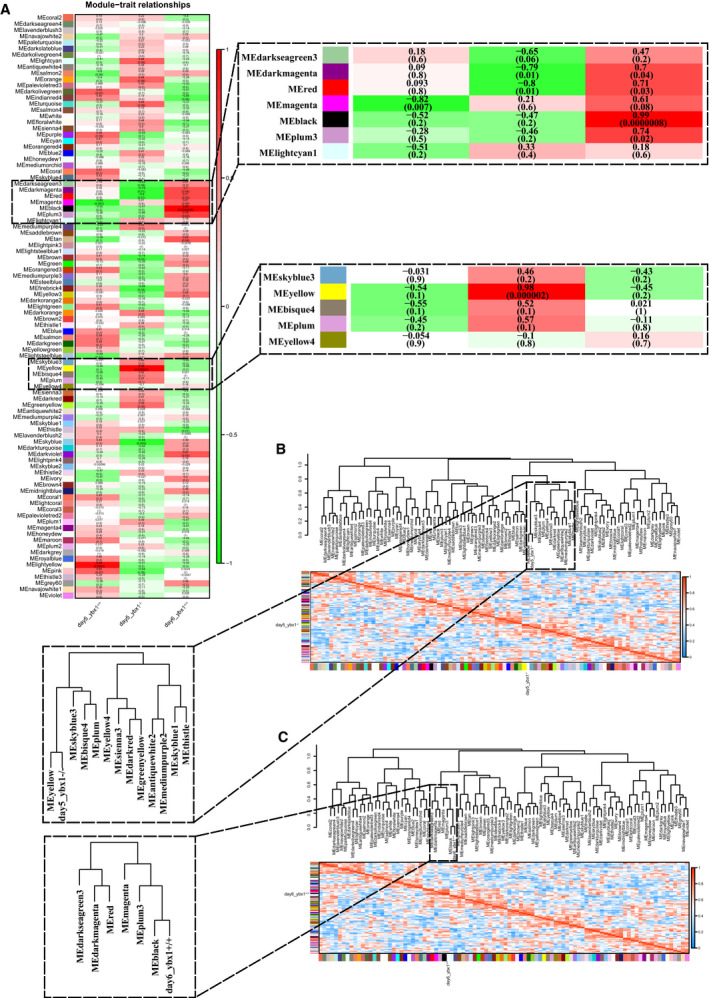
Detection of two trait‐related modules. (A) Module–trait associations. Each row corresponds to a module eigengene, and each column to a trait. Each cell displays the corresponding correlation and *P*‐value. (B) The eigengene dendrogram and heatmap identify that the yellow module is highly related to day5_*ybx1* ^−/−^. (C) The eigengene dendrogram and heatmap identify that the black module is highly related to day6_*ybx1* ^+/+^.

To investigate the lncRNAs, as well as their possible roles, implicated in Ybx1 gene knockout, we conducted a systematic coexpression network analysis using wgcna in r. Initially, the flashClust tools package was used to conduct cluster analysis on these samples to detect outliers; the results showed that all samples are in the clusters and passed the cuts (Fig. S5). Then a network‐topology analysis was conducted for several soft‐thresholding powers to have relative balance scale independence and mean connectivity of the wgcna. The lowest power for the scale‐free topology fit index was selected for construction of a hierarchical clustering tree (Fig. S6). This analysis colocalizes both correlated lncRNAs and mRNAs into 95 modules (Fig. S7). Each module contains independent datasets of transcripts (Table 4). The interactions among those modules were visualized in Fig. S8. The modules with common expression patterns that were significantly associated with specific traits (day5_*ybx1^−/−^* and day6_*ybx1^+/+^*) were detected based on the correlation between module eigengene and traits. Then the function plotEigengeneNetworks of wgcna was used to identify groups of correlated eigengenes. The results indicated that two modules (yellow and black) were significantly correlated to the traits: day5_*ybx1^−/−^* and day6_*ybx1^+/+^*, respectively (Fig. 3). The relationship of transcript significance with module membership was visualized in Fig. S9. The yellow modules significantly related to day5_*ybx1^−/−^* include 2394 mRNAs and 352 lncRNAs, whereas black modules correlated to day6_*ybx1^+/+^* contain 1075 mRNAs and 155 lncRNAs (Table 4).

**Table 4:** The number of mRNAs and lncRNAs in the top 10 modules.

Module	Size	lncRNA	mRNA
Turquoise	4731	318	4413
Blue	4284	328	3956
Brown	2752	382	2370
Yellow	2746	352	2394
Green	2267	355	1912
Red	1563	176	1387
Black	1230	155	1075
Pink	1206	207	999
Magenta	1109	98	1011
Purple	970	201	769

**Fig. 4:**
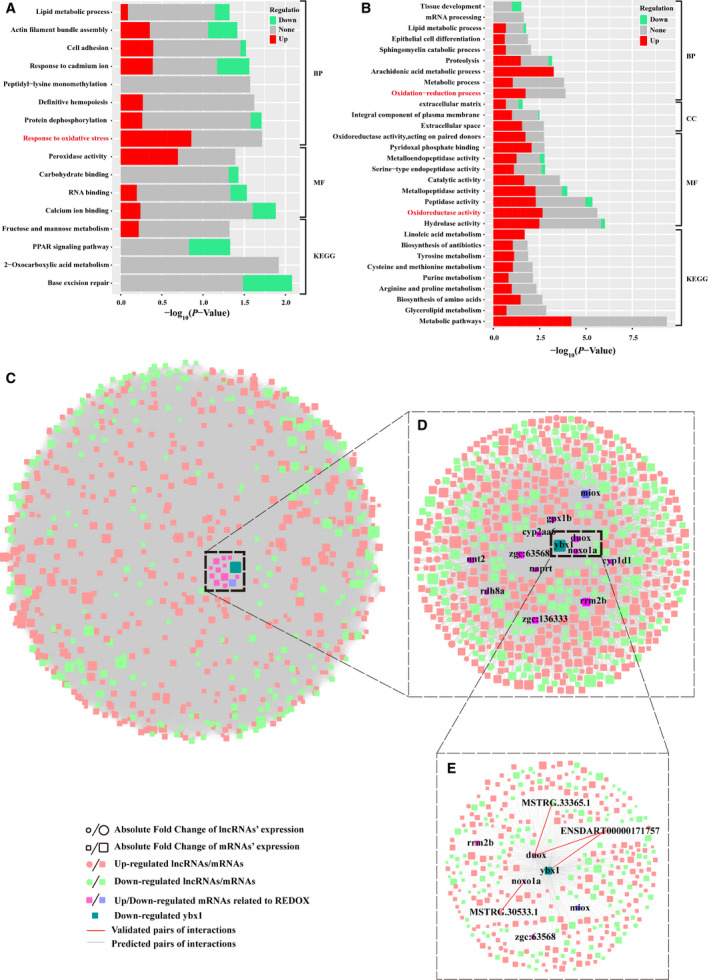
Coexpression network analysis defined REDOX‐related interactome in day5_*ybx1* ^−/−^‐related module. GO functional enrichment analysis of two trait‐related modules: (A) day5_*ybx1* *^−/−^*‐related module and (B) day6_*ybx1* ^+/+^‐related module. The colored bar reports the fraction of up‐regulated and down‐regulated transcripts present in that functional category. All the adjusted statistically significant values of the terms were negative 10‐base log transformed. KEGG, Kyoto Encyclopedia of Genes and Genomes; BP, Biological Process; MF, Molecular Function; CC, Cellular Component (C) Cytoscape map of the interaction (including mRNA–mRNA, mRNA–lncRNA and lncRNA–lncRNA) network defined by DE lncRNAs and mRNAs using the function *TOMsimilarityFromExpr* of wgcna package. (D) A zoomed view of a core network displaying interactions (including mRNA–mRNA and mRNA–lncRNA) involved in REDOX‐related genes and YBX1. (E) Zoomed in subnetwork view of interactions involved in *ybx1* and two key REDOX‐related genes: *duox*, *noxo1a*.

GO enrichment analyses based on the DAVID server were subsequently performed using the annotated transcripts (mRNAs) derived from two trait‐related modules. Unsurprisingly, the results indicated that many REDOX processes were significantly enriched by up‐regulated transcripts in two trait‐related modules with considerably high significance (highlighted in red text in Fig. 4A,B), including GO: 0006979 (response to oxidative stress), GO: 0016491 (oxidoreductase activity) and GO: 0055114 (oxidation–reduction process), etc. This finding is consistent with the results of functional enrichment analysis using DE RNAs described in the previous section (Fig. 2D), which further suggests that the decline of Ybx1 can enhance up‐regulation of REDOX. Indeed, many studies [29, 30, 31] have illustrated the influence of oxidative stress to embryonic development in zebrafish, e.g., oxidative stress condition could change the cellular REDOX state, which further causes oxidation of molecules, such as membrane peroxidation, loss of ions, protein cleavage and DNA strand breakages, and finally leads to cell death.

Next, to explore possible roles of lncRNAs in REDOX regulation triggered by decreased *y* *bx1*, we used the expression profile of DE lncRNAs and DE mRNAs included in the yellow module related to day5_*ybx1^−/−^* to assess the intramodular connectivity (including mRNA–mRNA and mRNA–lncRNA interactions) using TOM similarity, which was done by the function *TOMsimilarityFromExpr* of wgcna package. We did not further investigate the lncRNAs implicated in REDOX in black module related to day6_*ybx1^+/+^* because our previous DE analysis showed that just two DE lncRNAs were found in the comparison of day5_*ybx1^+/+^* versus day6_*ybx1^+/+^*. Regarding the yellow module, we detected a great number of interactions that involved 631 DE mRNAs and 45 DE lncRNAs (Fig. 4C). Among them, several lncRNAs, as well as mRNAs, were found to interact with two REDOX‐related mRNAs, that is, *duox* and *noxo1a* (Fig. 4D). These two genes have been demonstrated to play important roles in response to oxidative stress [32, 33]. Particularly, we observed that several lncRNAs not only exhibit strong correlations to *y* *bx1* but can also interact with two REDOX‐related mRNAs: *Duox* and *Noxo1a* (Fig. 4E).

### Correlations of lncRNA ENSDART00000171757 with embryonic development

**Fig. 5:**
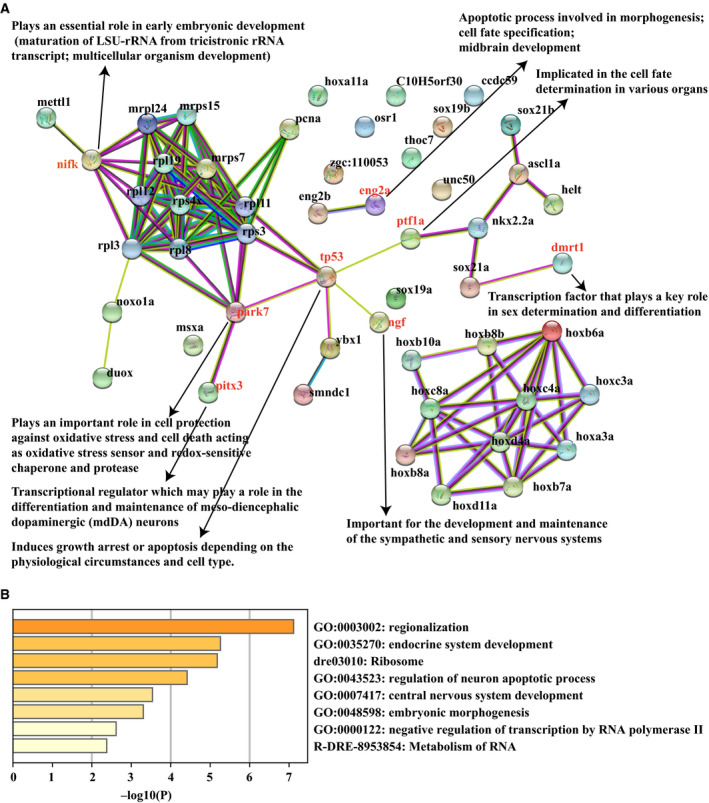
Prediction of lncRNA–protein interactions reveals close association of lncRNA ENSDART00000171757 with zebrafish embryonic development. (A) The lncRNA–protein interaction network derived from the STRING database. LSU‐rRNA, Large SubUnit (LSU) ribosomal RNA (rRNA). (B) Functional enrichment analysis of the possible interacted proteins in Metascape server.

Based on the previous network analysis, several lncRNAs associated with embryonic development, as well as the REDOX process, have been figured out. However, the underlying mechanism is yet unclear. For this purpose, a large‐scale screening of lncRNA–protein interactions was conducted based on the Ybx1‐related, REDOX‐related lncRNAs (displayed in Fig. 4E) via the *cat*RAPID server. We observed that the lncRNA ENSDART00000171757 exhibited moderate interactions with several regulatory proteins (Table S4), including transcription factors, RNA‐binding protein, etc. Subsequent functional analysis achieved by Metascape indicated that those proteins significantly enriched in diverse development processes (Fig. 5B), including regionalization, endocrine system development, central nervous system development, embryonic morphogenesis, etc. In addition, through screening their possible targets via a PPI (protein–protein interaction) database STRING, we found that those proteins cooperated with Ybx1, and two key REDOX‐related proteins, duox and noxo1a, might participate in a complex regulatory network involved in many developmental processes (Fig. 5A). Notably, nifk (ranked among top 3 proteins that interacted with lncRNA ENSDART00000171757 in *cat*RAPID) was found to interact with many ribosomal proteins and might act as a bridge to connect duox, noxo1a and ybx1 in the PPI network (Fig. 5B). Furthermore, it has been shown to play an essential role in early zebrafish embryonic development (insertional mutagenesis in zebrafish rapidly identifies genes essential for early vertebrate development). Our analysis indicated that knockout of *ybx1* in zebrafish might affect the expression of p53 and further disrupt the balance of the whole regulatory network and the expression of many key proteins involved in development, as well as lncRNA ENSDART00000171757, which might lead to overexpression of the REDOX‐related genes, that is, duox. Overexpression of REDOX‐related genes might further generate too much reactive oxygen and reactive nitrogen species (ROS/RNS), which result in oxidative stress and cause either cell death or senescence. These processes finally affect the zebrafish embryonic development.

### Bioassay validations

To test and confirm our findings, we subjected the lncRNA ENSDART00000171757 to an experimental validation study. Meanwhile, another two novel lncRNAs, MSTRG.30533.1 and MSTRG.33365.1, were selected for the same experiments, which also exhibit relatively significant differential expression in day5_*ybx1^+/+^* compared with day5_*ybx1^−/−^*, and simultaneously have strong correlations with *Duox* and *Noxo1a*, as well as *Ybx1*, in the network (Fig. 4).

#### Real‐time quantitative PCR validated five DE lncRNAs

For the real‐time quantitative PCR validation, we selected five lncRNAs, including three earlier mentioned lncRNAs, as well as another two lncRNAs, MSTRG.12630.1 and MSTRG.24792.1, which have relatively high fold change in differential analysis between day5_*ybx1^+/+^* and day5_*ybx1^−/−^* (Fig. S10). The expressions of MSTRG.12630.1, MSTRG.24792.1, ENSDART00000171757 and MSTRG.30533.1 were all apparently increased in day5_*ybx1^−/−^* larvae (Fig. S10A–D), and MSTRG33365.1 presented a sharp decrease in day5_*ybx1^−/−^* larvae (Fig. S10E). All the real‐time quantitative PCR results were consistent with the differential expression analysis.

#### Three REDOX‐related lncRNAs tend to be expressed in the gut

**Fig. 6:**
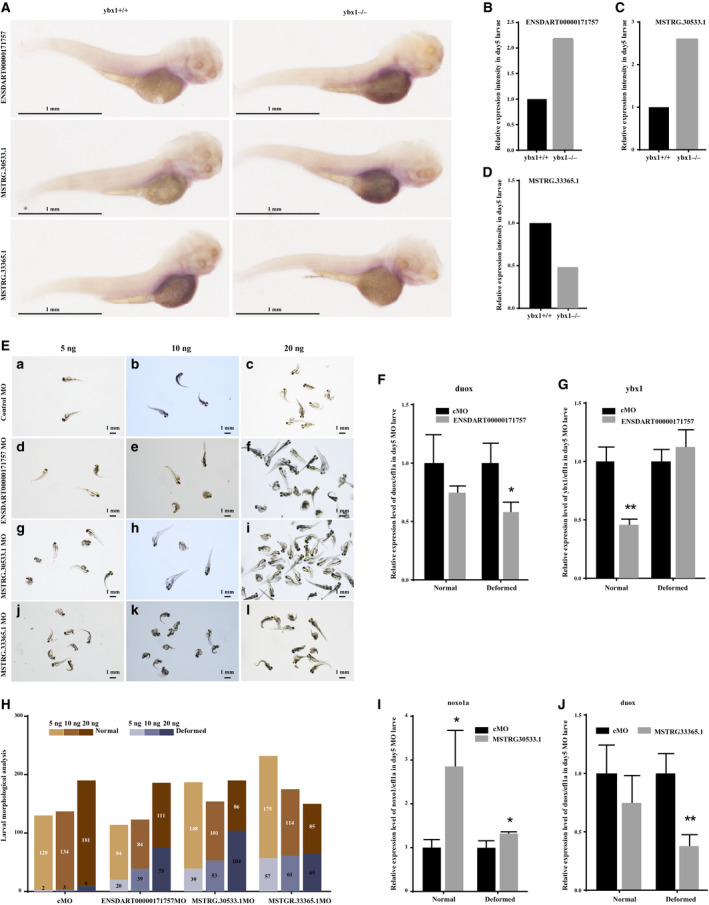
lncRNA knockdown led to larvae morphological deformation. (A) Whole‐mount *in situ* hybridization analysis of the three selected lncRNAs (ENSDART00000171757, MSTRG.30533.1 and MSTRG.33365.1) in day 5 *ybx1* ^+/+^ and *ybx1^−/−^* larvae. Top: the expression of ENSDART00000171757 lncRNA in day 5 *ybx1* ^+/+^ and *ybx1^−/−^* larvae. Middle: the expression of MSTRG.30533.1 lncRNA in day 5 *ybx1* ^+/+^ and *ybx1^−/−^* larvae. Bottom: the expression of lncRNA MSTRG.33365.1 in day 5 *ybx1* ^+/+^ and *ybx1^−/−^* larvae. Scale bars: 1 mm. (B–D) Relative expression intensity of ENSDART00000171757, MSTRG30533.1 and MSTRG33365.1 in day 5 *ybx1* ^+/+^ and *ybx1^−/−^* larvae. (E) Larval morphological analysis after lncRNA morpholino injection on day 5. (a–l) Photographs of deformed larvae injected with different doses (5, 10 and 20 ng) of morpholino for lncRNA (ENSDART00000171757, MSTRG.30533.1 and MSTRG.33365.1), and control morpholino injected larvae were used as reference. Scale bars: 1 mm. (F,G) Relative expression of *duox* mRNA and *ybx1* mRNA in day 5 larvae after injection of 20 ng ENSDART00000171757 morpholino. (H) Larval morphological statistics for different lncRNA morpholino larvae injected with different doses (5, 10 and 20 ng); control morpholino injection was used for reference. Column in brown represents larvae with normal morphology, and purplish blue column stands for deformed morphological larvae. (I) Relative expression of *noxo1a* mRNA in day 5 larvae after injection of 20 ng MSTRG.30533.1 morpholino. (J) Relative expression of *duox* mRNA in day 5 larvae after 20 ng MSTRG.33365.1 morpholino injection. 20 ng control morpholino injected larvae was used as reference. On day 5, morpholino‐injected larvae were divided into two groups, normal and deformed morphology. Columns in black represent control morpholino (cMO), and gray columns stand for targeted lncRNA morpholino. *P < 0.05, **P < 0.01. Error bars indicate standard deviation. Wilcox test was used to determine significance or lack thereof. The experiments were replicated three times.

We next tested whether the expression of three lncRNAs (ENSDART00000171757, MSTRG30533.1 and MSTRG.33365.1) has tissue specificity by whole‐mount *in situ* hybridization assay. Interestingly, we observed that all three lncRNAs were predominantly expressed in the gut region (Fig. 6A). Moreover, the expression level of those lncRNAs in day5_*ybx1^+/+^* and day5_*ybx1^−/−^* larvae was consistent with the real‐time quantitative PCR results (Fig. 6B–D).

#### lncRNA knockdown leads to larvae morphological deformation

Our network and PPI analysis revealed a lncRNA ENSDART00000171757 might be associated to zebrafish embryonic development. To test it, we targeted the lncRNA ENSDART00000171757, as well as other novel lncRNAs MSTRG.30533.1 and MSTRG.33365.1 (also exhibit strong correlations with *Duox* and *Noxo1a*, as well as *Ybx1*, in the network analysis), for morpholino knockdown, with their specific target sequence for morpholino synthesis. After serial morpholino injection (5, 10 and 20 ng), partial larvae on day 5 with severe morphological deformation were photographed (Fig. 6E). As a result, a large proportion of deformed larvae was observed for each lncRNA’s morpholino, but only a few deformed larvae by control morpholino injection. In particular, the rate of larval deformation after morpholino injection was dose dependent for all three lncRNAs (Fig. 6H). Our experiments showed that the knockdown of each of the three selected lncRNAs leads to larvae morphological deformation in a dose‐dependent manner.

#### Correlation of lncRNAs with *duox*, *noxo1a* and *ybx1*

Our network analysis indicated that the lncRNAs ENSDART00000171757, MSTRG.30533.1 and MSTRG.33365.1 exhibited strong correlations to the REDOX‐related genes *duox* and *noxo1a*. To validate it, we divided larvae (day 5) injected with 20 ng morpholino for the three lncRNAs into two groups, normal morphology and deformed morphology. We observed that knockdown of ENSDART00000171757 slightly reduced the mRNA expression of *duox* in both the normal and the deformed groups (Fig. 6F) and remarkably reduced the mRNA expression of *ybx1* in the normal group (Fig. 6G). MSTRG.30533.1 knockdown apparently activated *noxo1a* mRNA expression in the normal group (Fig. 6I), whereas inhibition of MSTRG.33365.1 led to a sharp decrease of *duox* mRNA expression in the deformed group (Fig. 6J). Our experiments demonstrated that both lncRNAs ENSDART00000171757 and MSTRG.33365.1 might positively regulate the expression of *duox*, whereas MSTRG30533.1 probably exhibits negative regulation to *noxo1a*. lncRNA ENSDART00000171757 also exhibited positive regulation to *y* *bx1*.

Also, we tested the potential interaction between ENSDART00000171757 and Ybx1 in protein level. Immunoblotting was performed to evaluate Ybx1 protein expression level under ENSDART00000171757 morpholino knockdown, and beta‐actin was used as an internal reference. The results showed that the expression of Ybx1 protein was evidently reduced after ENSDART00000171757 knockdown (Fig. S11A). It was suggested that inhibition of ENSDART00000171757 would result in significant reduction (approximately 50%) of the relative intensity of Ybx1/Actin in day 5 larvae (Fig. S11B).

## Discussion

Given that lncRNAs have been proposed to carry out diverse functions, for instance, regulation of gene expression by acting as signaling, guiding, sequestering or scaffolding molecules, an increasing number of studies focused on the disclosure of the roles of lncRNA in vertebrate development [34, 35, 36]. In some cases, however, similar works on the same lncRNAs come to contradictory conclusions. A typical example is that Lin *et al*.’s study [37] and Ulitsky *et al*.’s study [38] showed that knockdown of the lncRNA megamind led to zebrafish embryonic defects, whereas Kok *et al*.’s study [39] demonstrated that megamind had no influence on embryonic development. These inconsistent results have caused a dispute about whether lncRNA is important to vertebrate development. Notably, Goudarzi *et al*.’s study [14] used the CRISPR/Cas9 system to knock out 24 specific lncRNAs in zebrafish, which was picked out based on synteny, conservation, expression dynamics and proximity to development‐related genes and concluded that individual lncRNAs have no overt functions in zebrafish embryogenesis, viability and fertility. Given these facts, it is likely that lncRNAs exert limited functions in vertebrate development, particularly in zebrafish embryonic development. Ybx1 is an important protein to embryogenesis and development, disclosure of the correlation between *y* *bx1* and lncRNAs transcriptome will shed new insights for unveiling organelle‐based mechanisms of lncRNAs implicated in zebrafish embryo development. We believe that our study also might be regarded as complementary research to provide some clues for the issue whether the lncRNAs are important to vertebrate development.

Worthy of a special mention is the fact that despite that the RNA library in this study is prepared using oligo‐dT to capture sequences with poly‐A tail, a great amount of lncRNAs could still be sequenced. The reason is that lncRNAs are structurally similar to mRNAs in some way, that is, the majority of mature lncRNAs are produced by the same RNA polymerase II transcriptional machinery and can be polyadenylated, suggesting some of them are indistinguishable from mRNAs in structure. In this study, we reconstructed the zebrafish transcriptome and identified a high‐confidence dataset of lncRNAs using a stringent filtering pipeline. Next, to figure out the lncRNAs implicated in zebrafish embryonic development, we performed a series of systematic transcriptomic analyses.These analyses concluded that decreasing *ybx1* by acquired knockout or innate autoregulation exhibits close correlations to REDOX processes that involved several lncRNA regulations.

Embryo development is a complicated process that underlies precise regulation and control. The roles of REDOX in embryonic development have been well elucidated so far. Concretely, oxygen can be regarded as a double‐edged sword because it is essential to embryogenesis but also acts as a potential hazard via formation of ROS/RNS [40]. Too much ROS leads to oxidative stress and further causes either cell death or senescence by oxidation of cellular molecules [41]. REDOX balance is thus critical to embryonic development. Notably, ROS generated by NADPH oxidases is a crucial REDOX signal to establish homeostasis [32]. This knowledge directs us to narrow down the huge number of mRNA–lncRNA interactions to a relatively small subnetwork exhibiting strong correlation with the two key REDOX‐related genes, *duox* and *noxo1a*. Among them, analysis of lncRNA–protein interactions next figured out a lncRNA (ENSDART00000171757) might interact with many proteins, which has already been demonstrated to be associated to various zebrafish embryonic development processes. For instance, nifk encodes a protein that is important to early embryonic development. The protein ang2a was found to be involved in the apoptotic process of morphogenesis, cell fate specification and midbrain development. Park7 was shown to play an important role in cell protection against oxidative stress and cell death acting as an oxidative stress sensor and REDOX sensitive. It was implied that the lncRNA might be a key molecule participating in a complex regulatory network involved in those critical proteins, that is, ybx1, noxo1a and duox, etc. Indeed, subsequent validation bioassays support our predictions. Certainly, further studies are needed to clarify more detailed mechanisms.

In conclusion, our systematic transcriptome analysis successfully disclosed many lncRNAs that might be important to zebrafish embryonic development, particularly in lncRNA ENSDART00000171757. This study provides new insights into a molecular mechanism of zebrafish embryonic development. This could also potentially pave a new way to investigate the functions of lncRNAs for the regulatory elements through binding to their respective partners during the development process.

## Conflict of interest

The authors declare no conflict of interest.

## Author contributions

CH and BZ designed the research and wrote the manuscript. CH and DL made bioinformatics analysis. BZ validated the experiments. XDZ and WG reviewed the manuscript and supervised the research. All authors read and approved the final manuscript.

## Supporting information


**Table S1.** Basic statistics of zebrafish RNA‐seq data before and after quality trimming.
**Table S2.** Basic statistics of assembly results of transcriptome in zebrafish.
**Table S3.** Basic statistics of quality assessment of assembled transcripts achieved by DETONATE.
**Table S4.** Potential interacted proteins of lncRNA ENSDART00000171757 predicted from *cat*RAPID server.
**Fig. S1.** Heatmap of DE transcripts detected in comparison between day5_ybx1^+/+^ and day5_ybx1*^−/−^*.
**Fig. S2.** Volcano plot of DE transcripts detected in comparison between day5_ybx1^+/+^ and day5_ybx1^−/−^.
**Fig. S3.** Heatmap of DE transcripts detected in comparison between day5_ybx1^+/+^ and day6_ybx1^+/+^.
**Fig. S4.** Volcano plot of differentially expressed transcripts detected in comparison between day5_ybx1^+/+^ and day6_ybx1^+/+^.
**Fig. S5.** Sample clustering to detect outliers.
**Fig. S6.** Analysis of network topology for various soft‐thresholding powers.
**Fig. S7.** Clustering dendrograms of transcripts, with dissimilarity based on topological overlap, together with assigned module colors.
**Fig. S8.** Visualizing the gene network using a heatmap plot.
**Fig. S9.** Scatterplots of Gene Significance (GS) for recurrence versus Module Membership (MM) in the yellow module (A) and black module (B).
**Fig. S10.** Reverse transcription and real‐time quantitative PCR to validation of DE lncRNA identified by RNA‐seq between day 5 *ybx1* ^+/+^ and *ybx1* ^−/−^ larvae.
**Fig. S11.** Immunoblotting study for validation of the correlation between Ybx1 expression and ENSDART00000171757 lncRNA knockdown.

## Data Availability

This Transcriptome Shotgun Assembly (TSA) project was deposited at Gene Expression Omnibus repository under the accession number GSE134334.
