# Supplementary material for: Long noncoding RNAs implicated in embryonic development in Ybx1 knockout zebrafish
Source: FEBS Open Bio. 2021 Feb 26;11(4):1259–76. doi: 10.1002/2211-5463.13057 (PMC8016120; doi:10.1002/2211-5463.13057)

Supplementary Material

Long noncoding RNAs implicated in embryonic development in Ybx1 knockout zebrafish

Chen Huang[1†], Bo Zhu[2†], Dongliang Leng[2], Wei Ge[2*], Xiaohua Douglas Zhang[2*]

[1]Stat Key laboratory of Quality Research in Chinese Medicine, Macau Institute For Applied Research in Medicine and Health, Macau University of Science and Technology, Macau, SAR, China

[2]Faculty of Health Sciences, University of Macau, Taipa, Macau

[†]These authors contributed equally to this work

[*]To whom correspondence should be addressed. Email: douglaszhang@um.edu.mo; weige@um.edu.mo

Table S1 Basic statistics of zebrafish RNA-seq data before and after quality trimming.

Sample ID	Genotype	Raw reads	Raw base	Clean reads	Clean base
Day5_1	$YBX1^{+/+}$	60,690,292	3,546,045,792	55,949,996	3,327,642,891
Day5_2	$YBX1^{+/+}$	79,155,512	4,704,205,704	75,522,326	4,491,354,563
Day5_3	$YBX1^{+/+}$	21,753,664	1,291,650,684	20,932,992	1,244,929,870
Day5M_1	$YBX1^{-/-}$	69,764,716	6,256,715,790	63,546,418	5,855,485,854
Day5M_2	$YBX1^{-/-}$	36,919,676	3,622,069,913	36,065,872	3,537,806,923
Day5M_3	$YBX1^{-/-}$	46,519,762	4,580,115,275	45,583,820	4,487,415,546
Day6_1	$YBX1^{+/+}$	34,156,800	2,032,720,058	32,494,270	1,932,357,536
Day6_2	$YBX1^{+/+}$	86,957,662	5,170,969,602	83,992,144	4,995,557,421
Day6_3	$YBX1^{+/+}$	31,001,772	3,055,951,587	30,330,530	2,987,673,962

Table S2 Basic statistics of assembly results of transcriptome in zebrafish.

**Statistics terms**	**Number**
Total number	77,252
Total length of (bp)	135,473,390
Average length (bp)	1,753
N50 Length (bp)	2,861
Maximum length (bp)	88,553
Minimum length (bp)	10
GC content (%)	41.75

Table S3. Basic statistics of quality assessment of assembled transcripts achieved by DETONATE.

weighted_nucl_precision	0.970402
weighted_nucl_recall	0.999876
weighted_nucl_F1	0.984919
unweighted_nucl_precision	0.864243
unweighted_nucl_recall	0.99868
unweighted_nucl_F1	0.926611
weighted_pair_precision	0.95342
weighted_pair_recall	0.996775
weighted_pair_F1	0.974615
unweighted_pair_precision	0.869981
unweighted_pair_recall	0.993726
unweighted_pair_F1	0.927745
weighted_contig_recall	0.999912
weighted_contig_precision	0.950629
weighted_contig_F1	0.974648
unweighted_contig_recall	0.999145
unweighted_contig_precision	0.801403
unweighted_contig_F1	0.889416
weighted_kmer_recall	0.999847
inverse_compression_rate	0.451578
kmer_compression_score	0.548269
weighted_kmer_KL_A_to_M	0.0100652
weighted_kmer_KL_B_to_M	0.0236129
weighted_kmer_jensen_shannon	0.0168391
weighted_kmer_hellinger	0.128502
weighted_kmer_total_variation	0.0336688
unweighted_kmer_KL_A_to_M	0.0358701
unweighted_kmer_KL_B_to_M	0.0844331
unweighted_kmer_jensen_shannon	0.0601516
unweighted_kmer_hellinger	0.242076
unweighted_kmer_total_variation	0.128099

Table S4 Potential interacted proteins of lncRNA ENSDART00000171757 predicted from catPAPID server.

ID[a]	Gene name	Protein name	Power[b]	Functions
Q8JIY8	nifk	MKI67 FHA domain-interacting nucleolar phosphoprotein	0.73	Plays an essential role in early embryonic development (maturation of LSU-rRNA from tricistronic rRNA transcript; multicellular organism development)
P09015	eng2a	Homeobox protein engrailed-2a	0.74	apoptotic process involved in morphogenesis; cell fate specification; midbrain development;
Q90470	hoxd11a	Homeobox protein Hox-D11a	0.67	Sequence-specific transcription factor which is part of a developmental regulatory system
Q9PTP1	pcna	Proliferating cell nuclear antigen	0.73	involved in the control of eukaryotic DNA replication
Q9PWL7	hoxb10a	Homeobox protein Hox-B10a	0.63	Sequence-specific transcription factor which is part of a developmental regulatory system
Q90259	ascl1a	Achaete-scute homolog 1a	0.75	Transcriptional regulator. Involved in neurogenesis. Required for the development of neurons
Q8JH55	hoxb8b	Homeobox protein Hox-B8b	0.78	Sequence-specific transcription factor which is part of a developmental regulatory system
P28174	hoxc3a	Homeobox protein Hox-C3a	0.75	Sequence-specific transcription factor which is part of a developmental regulatory system
Q7ZV80	smndc1	Survival of motor neuron-related-splicing factor 30	0.87	Necessary for spliceosome assembly.
Q6DGZ3	thoc7	THO complex subunit 7 homolog	0.75	Required for efficient export of polyadenylated RNA.
Q68EH7	hoxc8a	Homeobox protein Hox-C8a	0.72	Sequence-specific transcription factor which is part of a developmental regulatory system
Q03357	msxa	Homeobox protein MSH-A	0.71	Probable morphogenetic role
Q8AWZ0	hoxb8a	Homeobox protein Hox-B8a	0.77	Sequence-specific transcription factor which is part of a developmental regulatory system
Q6QB00	helt	Hairy and enhancer of split-related protein helt	0.64	Transcriptional repressor which binds preferentially to the canonical E box sequence.
P15861	hoxb6a	Homeobox protein Hox-B6a	0.86	Sequence-specific transcription factor which is part of a developmental regulatory system
Q6P5L3	rpl19	60S ribosomal protein L19	0.70	RNA binding
Q9DDU0	hoxa11a	Homeobox protein Hox-A11a	0.61	Sequence-specific transcription factor which is part of a developmental regulatory system
Q7ZSX3	ptf1a	Pancreas transcription factor 1 subunit alpha	0.66	Implicated in the cell fate determination in various organs

Q6DGL8	mrps15	28S ribosomal protein S15, mitochondrial	0.67	structural constituent of ribosome
O57374	hoxd4a	Homeobox protein Hox-D4a	0.85	Sequence-specific transcription factor which is part of a developmental regulatory system
Q9DDD7	sox19b	Transcription factor Sox-19b	0.70	Transcriptional activator
Q642H9	rps4x	40S ribosomal protein S4, X isoform	0.64	RNA binding
P79734	tp53	Cellular tumor antigen p53	0.63	Induces growth arrest or apoptosis depending on the physiological circumstances and cell type.
Q9PWM3	hoxc4a	Homeobox protein Hox-C4a	0.60	Sequence-specific transcription factor which is part of a developmental regulatory system
Q498Z6	mrps7	28S ribosomal protein S7, mitochondrial	0.80	mRNA binding
Q90481	nkx2.2a	Homeobox protein Nkx-2.2a	0.58	Transcriptional activator involved in the development of insulin-producting beta cells in the endocrine pancreas
Q7SZS1	sox21a	Transcription factor Sox-21-A	0.78	May function as a switch in neuronal development
Q6RVD7	sox21b	Transcription factor Sox-21-B	0.69	Acts as a negative regulator of transcription
Q6QU75	pitx3	Pituitary homeobox 3	0.66	Transcriptional regulator which may play a role in the differentiation and maintenance of meso-diencephalic dopaminergic (mdDA) neurons
Q6DH02	mrpl24	Probable 39S ribosomal protein L24, mitochondrial	0.65	structural constituent of ribosome
P31533	eng2b	Homeobox protein engrailed-2b	0.63	DNA binding
Q8AWY9	hoxb7a	Homeobox protein Hox-B7a	0.77	Sequence-specific transcription factor which is part of a developmental regulatory system
Q5XJ57	mettl1	tRNA (guanine-N(7)-)-methyltransferase	0.75	Methyltransferase that mediates the formation of N7-methylguanine in a subset of RNA species
Q5XJ36	park7	Parkinson disease protein 7 homolog	0.57	plays an important role in cell protection against oxidative stress and cell death acting as oxidative stress sensor and redox-sensitive chaperone and protease
Q5XJQ7	osr1	Protein odd-skipped-related 1	0.60	Regulates mesoderm versus endoderm differentiation
Q5RGP9	ccdc59	Thyroid transcription factor 1-associated protein 26 homolog	0.61	Reactome
Q503V9	endoub	Poly(U)-specific endoribonuclease-B	0.51	cleaves single-stranded RNAs

P47792	sox19a	Transcription factor Sox-19a	0.65	Transcriptional activator
B3DHS1	macir	Macrophage immunometabolism regulator	0.64	May play a role in immune regulation through regulation of the macrophage function
Q9PWL5	hoxa4a	Homeobox protein Hox-A4a	0.66	Sequence-specific transcription factor which is part of a developmental regulatory system
Q7ZUU1	unc50	Protein unc-50 homolog	0.61	May be involved in cell surface expression of neuronal nicotinic receptors.
Q71MM5	dmrt1	Doublesex- and mab-3-related transcription factor 1	0.57	Transcription factor that plays a key role in sex determination and differentiation
Q6YBR5	ngf	Nerve growth factor	0.59	important for the development and maintenance of the sympathetic and sensory nervous systems

Figure S1. Heatmap of differentially expressed transcripts detected in comparison between day5_ybx1$^{+/+}$ vs day5_ybx1$^{-/-}$.

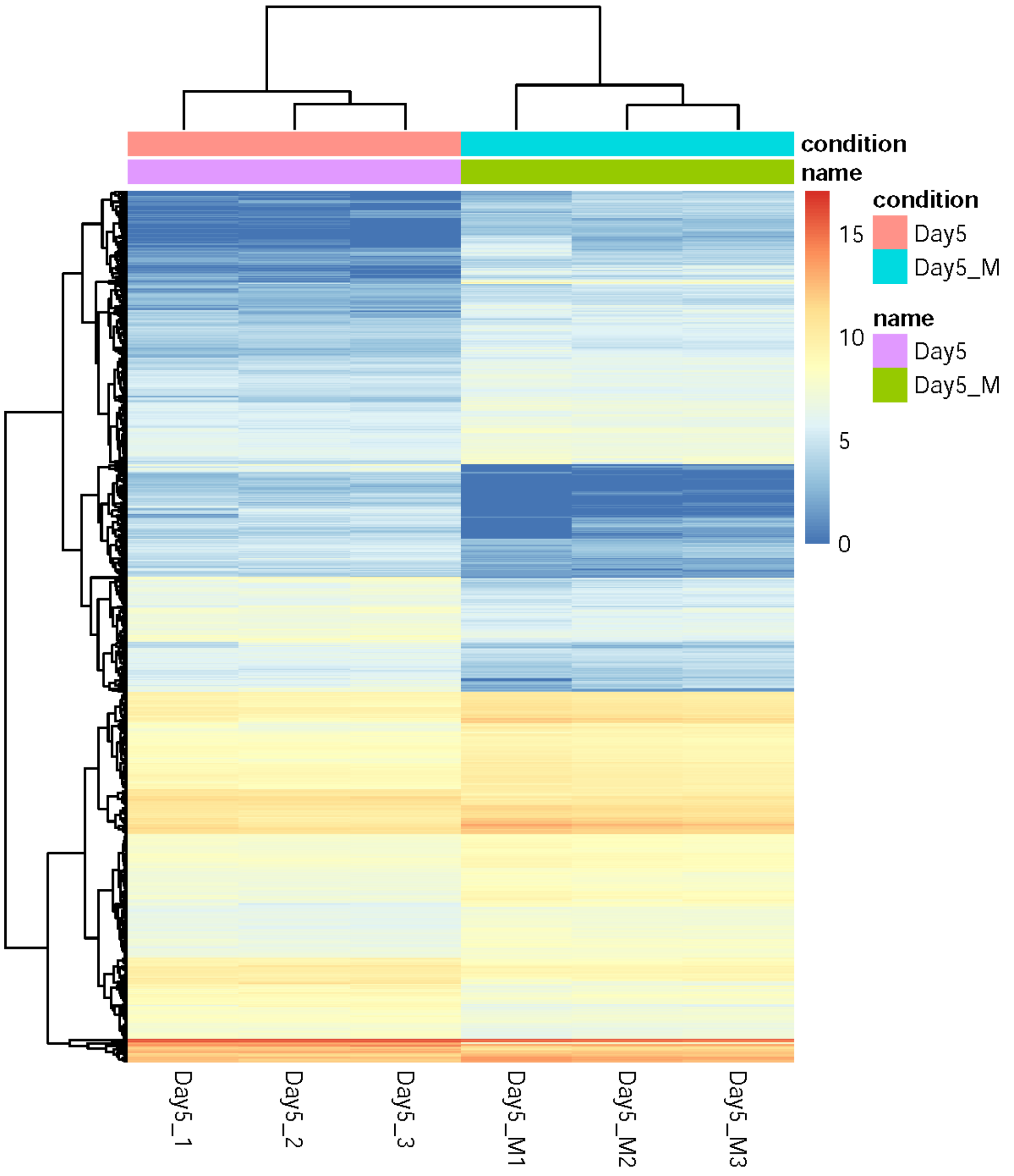

Figure S2. Volcano Plot of differentially expressed transcripts detected in comparison between day5_ybx1$^{+/+}$ vs day5_ybx1$^{-/-}$. Each spot represents a transcript. Red spots represent differentially expressed transcripts.

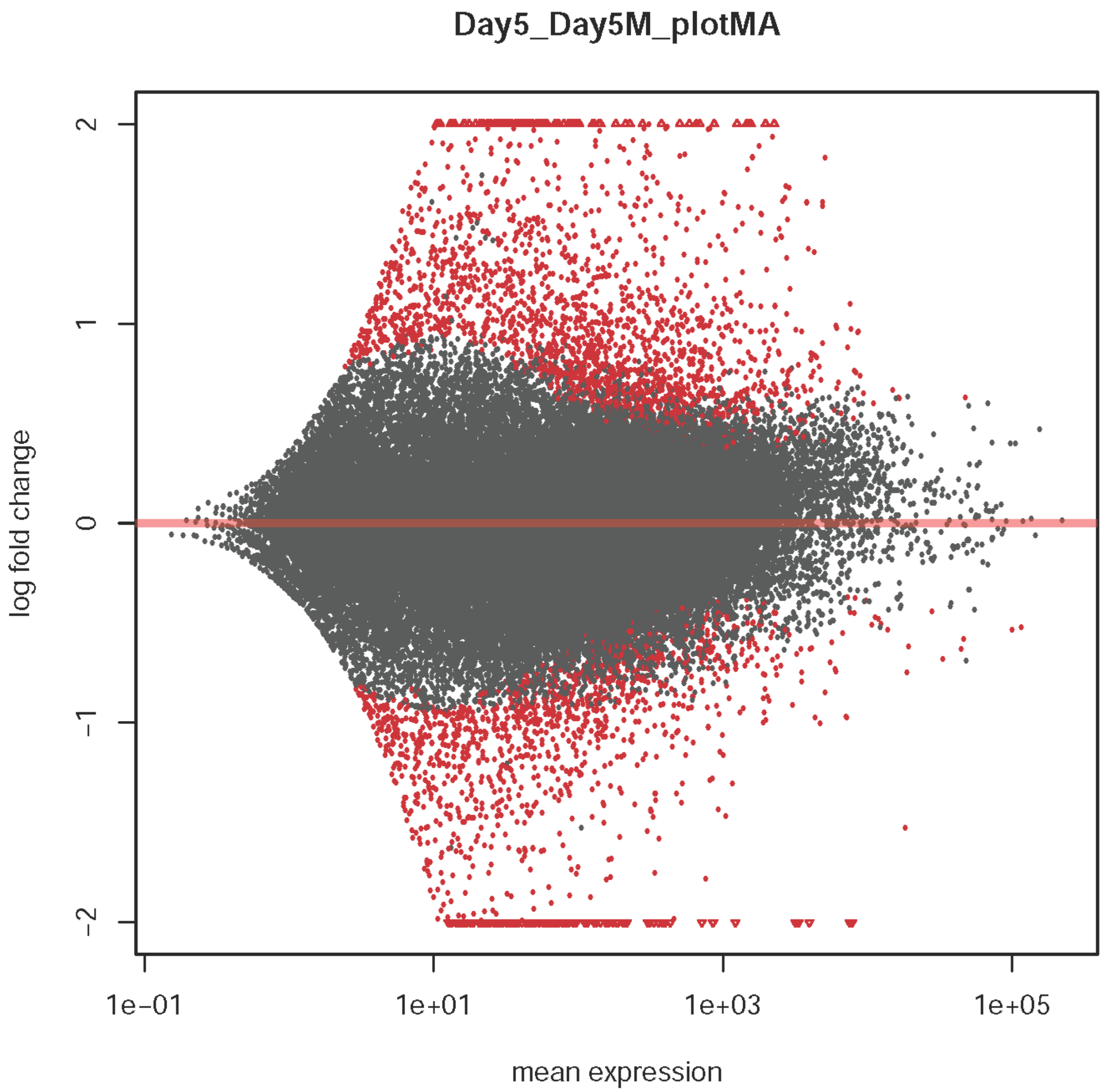

Figure S3. Heatmap of differentially expressed transcripts detected in comparison between day5_ybx1$^{+/+}$ vs day6_ybx1$^{+/+}$.

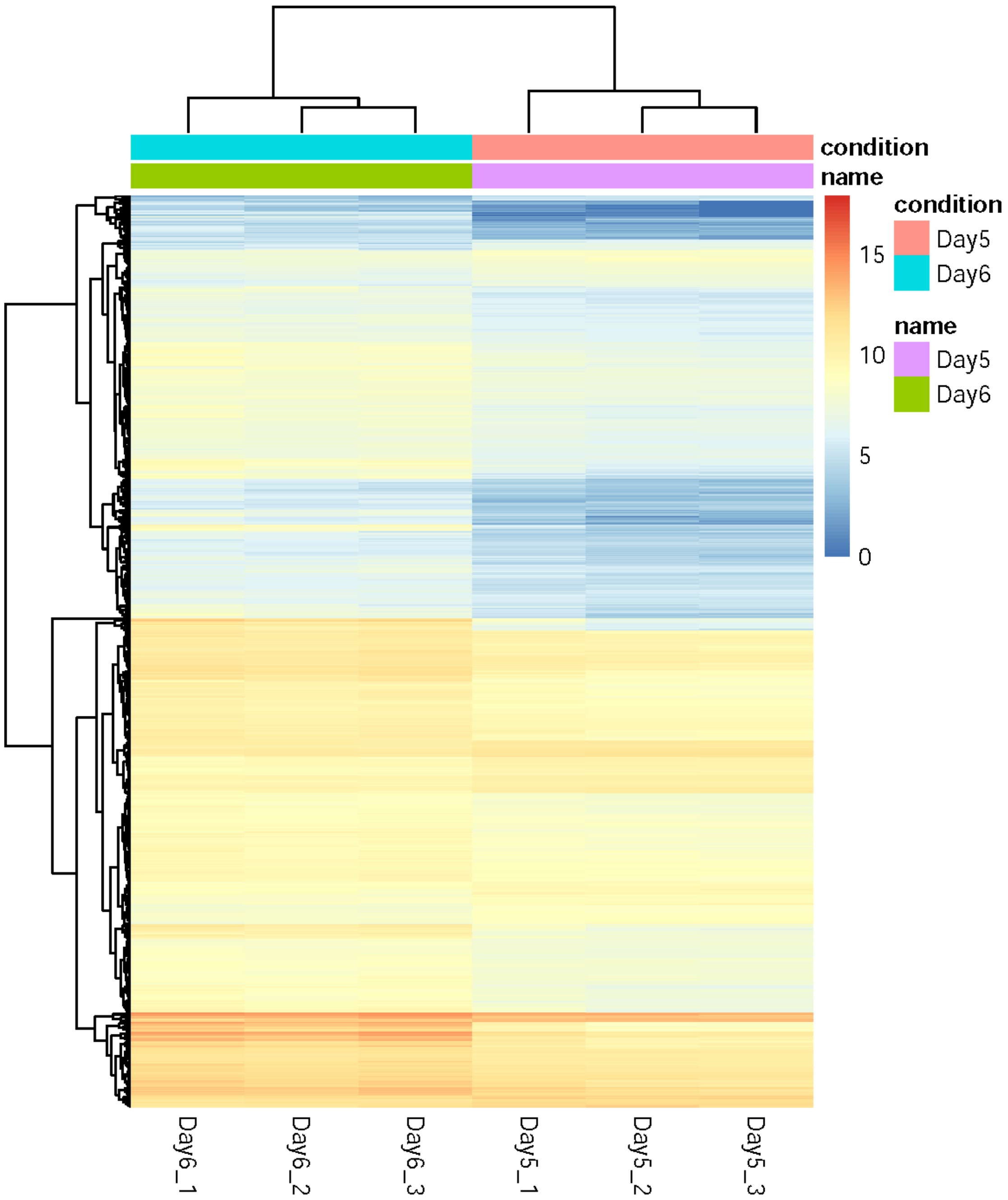

Figure S4. Volcano Plot of differentially expressed transcripts detected in comparison between day5_ybx1$^{+/+}$ vs day6_ybx1$^{+/+}$. Each spot represents a transcript. Red spots represent differentially expressed transcripts.

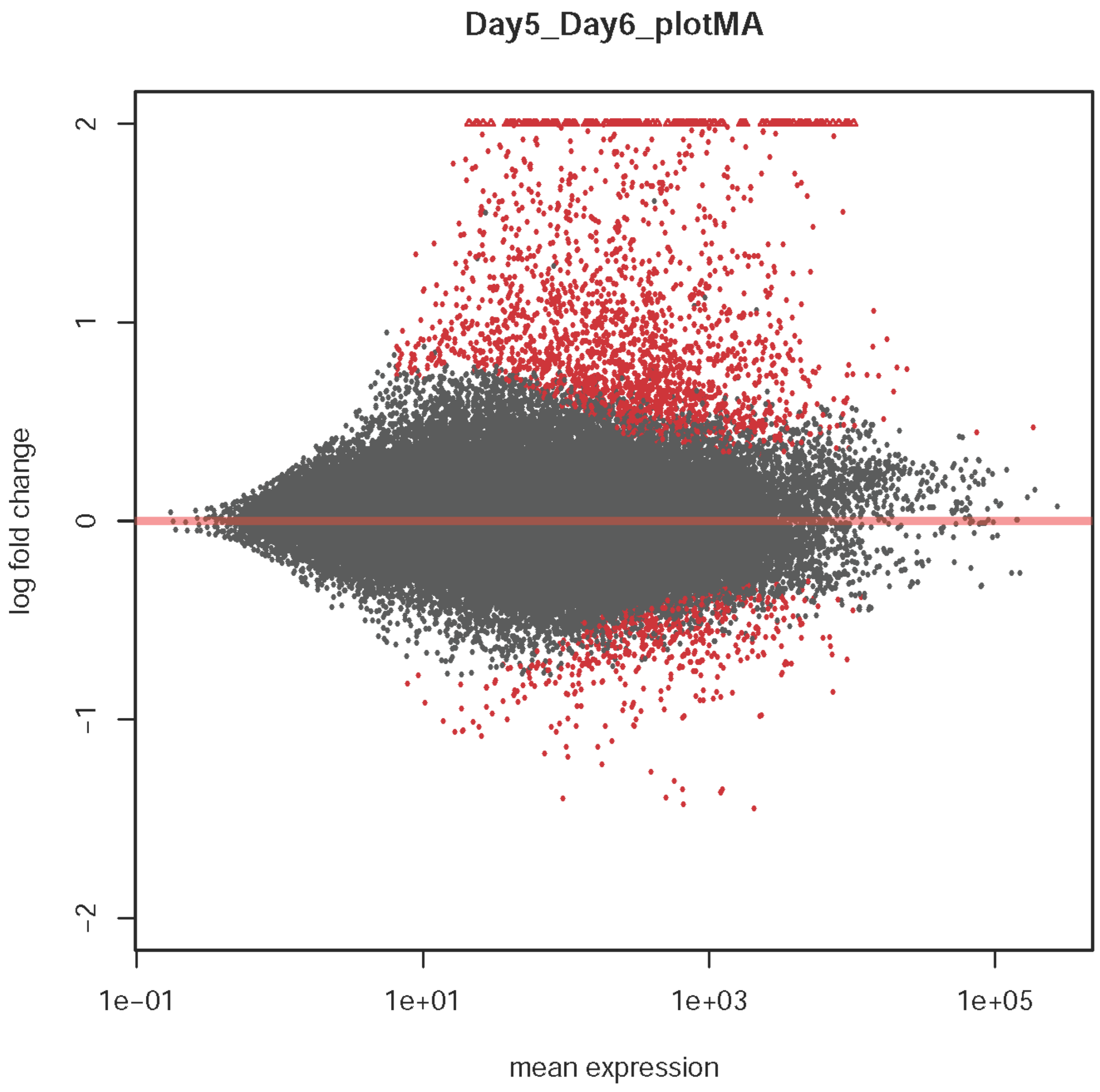

Figure S5. Sample clustering to detect outliers. All the samples were in the clusters, all samples have passed the cuts.

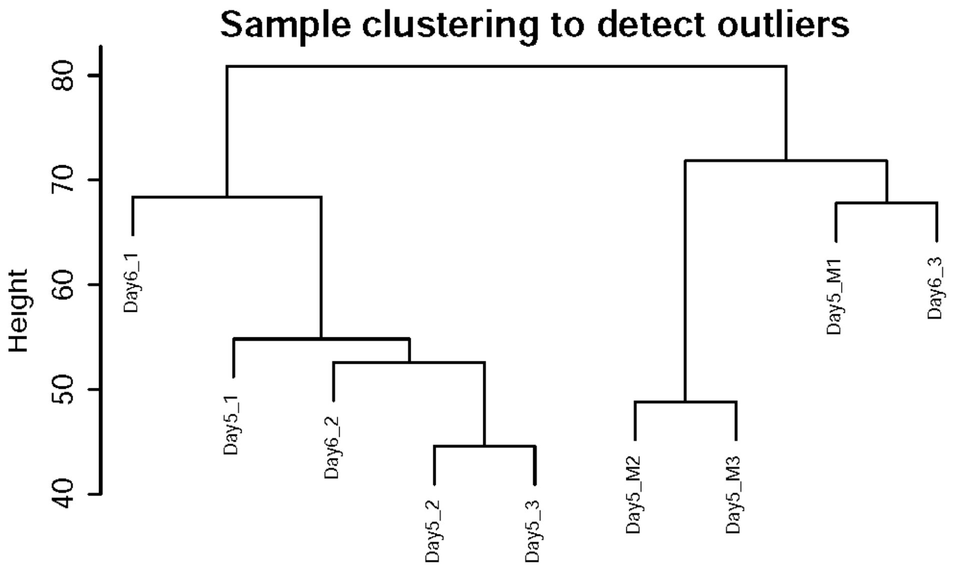

Figure S6. Analysis of network topology for various soft-thresholding powers. The left panel indicates the scale-free fit index (y-axis) as a function of the soft-thresholding power (x-axis). The right panel shows the mean connectivity (degree, y-axis) as a function of the soft-thresholding power (x-axis).

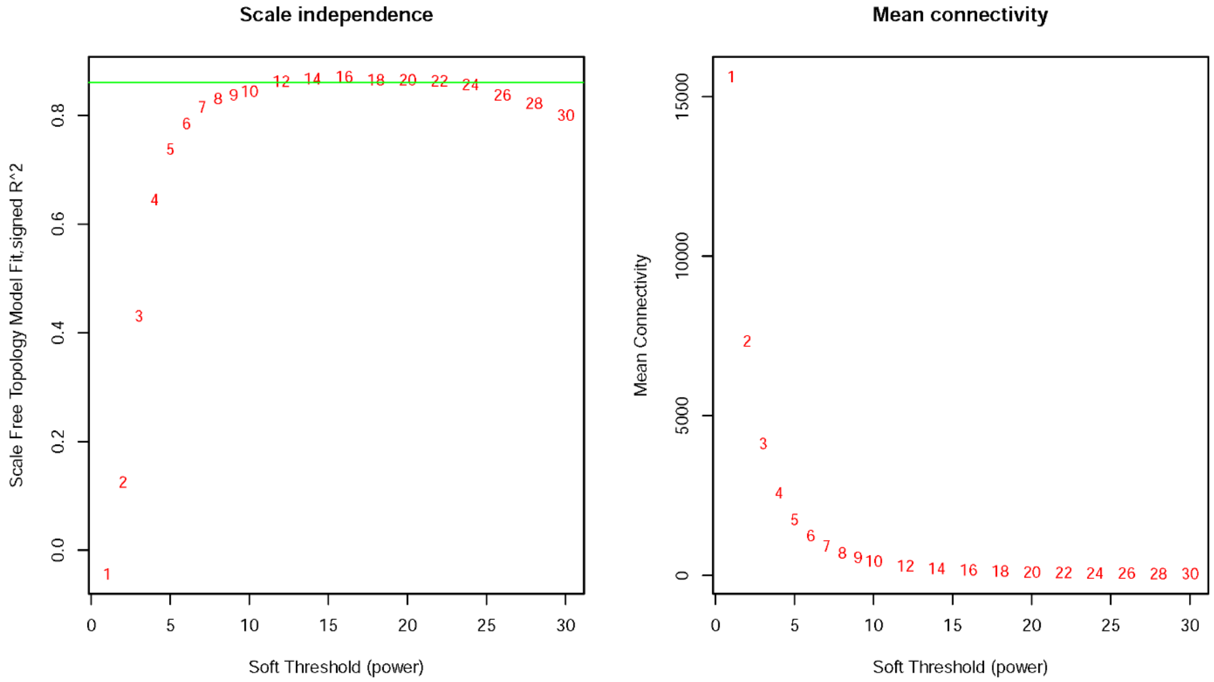

Figure S7. Clustering dendrograms of transcripts, with dissimilarity based on topological overlap, together with assigned module colors.

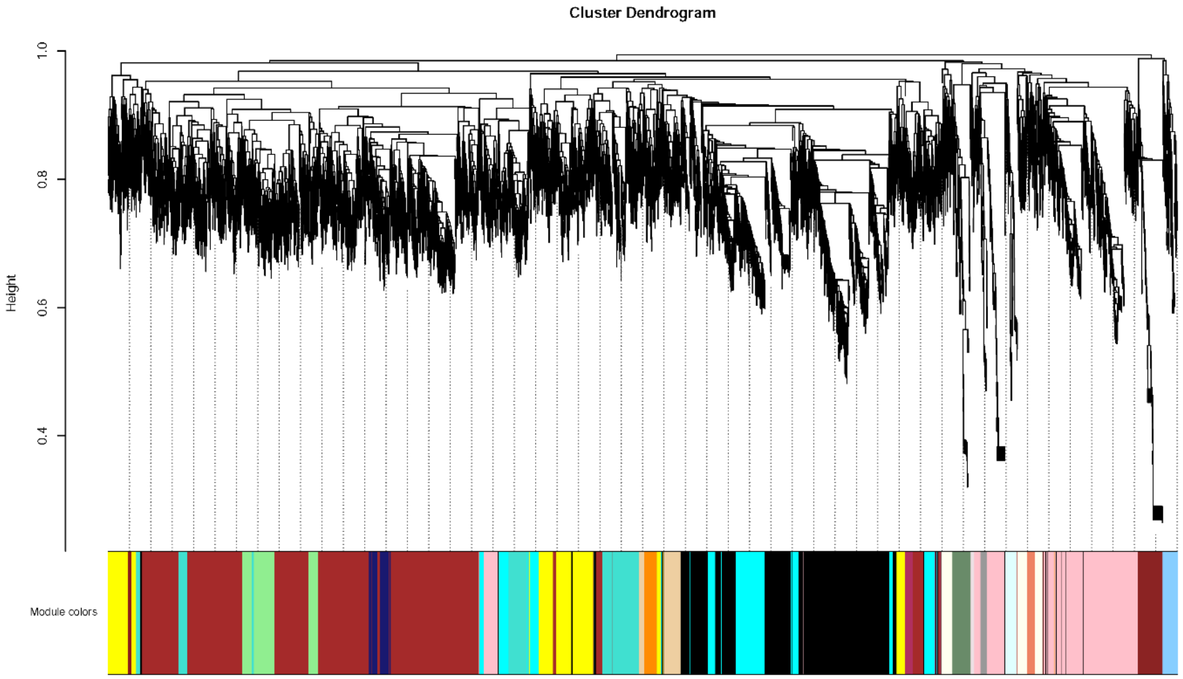

Figure S8. Visualizing the gene network using a heatmap plot. Light color represents low overlap and progressively darker red color represents higher overlap.

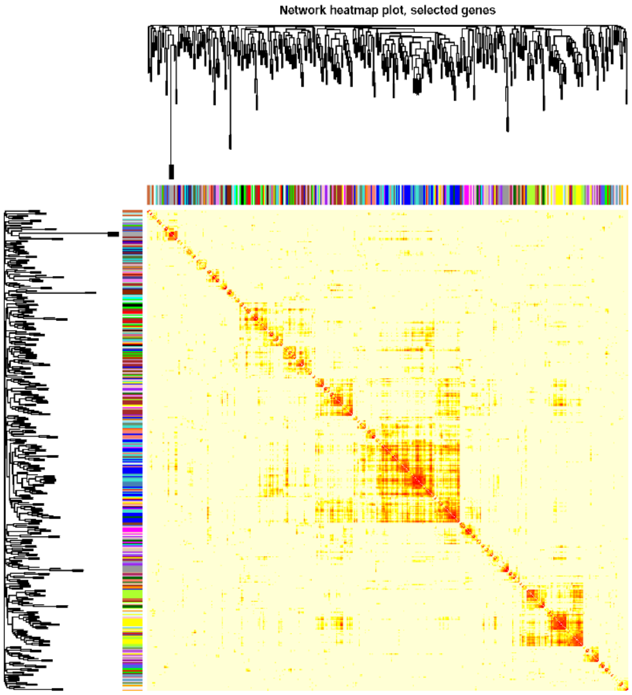

Figure S9. Scatterplots of Gene Significance (GS) for recurrence vs Module Membership (MM) in the yellow module (A) and black module (B).

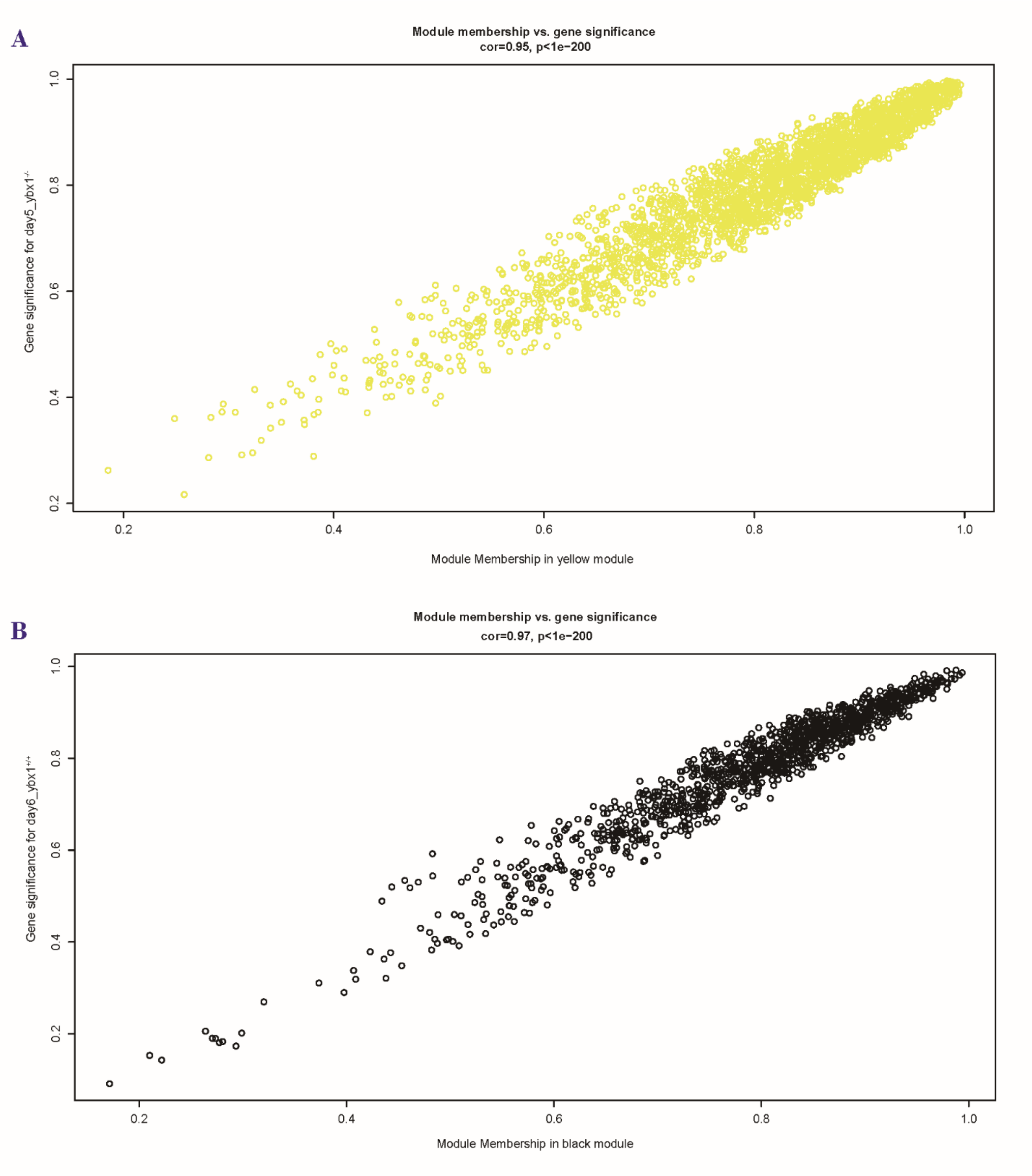

Figure S10. Reverse transcription and real-time quantitative PCR to validation of differentially expressed lncRNA identified by RNA-seq between day5 *ybx1* $^{+/+}$ and *ybx1* $^{-/-}$ larvae. A-E: Relative expression level of five selected differential lncRNA in both day5 *ybx1* $^{+/+}$ and *ybx1* $^{-/-}$ larvae, they are MSTRG12630.1, MSTRG24792.1, ENSDART00000171757, MSTRG30533.1, MSTRG33365.1 respectively. *:p value <0.05, **:p value<0.01, ***:p value<0.001. Wilcox test used to determine significance or lack thereof. The experiments replicates three times.

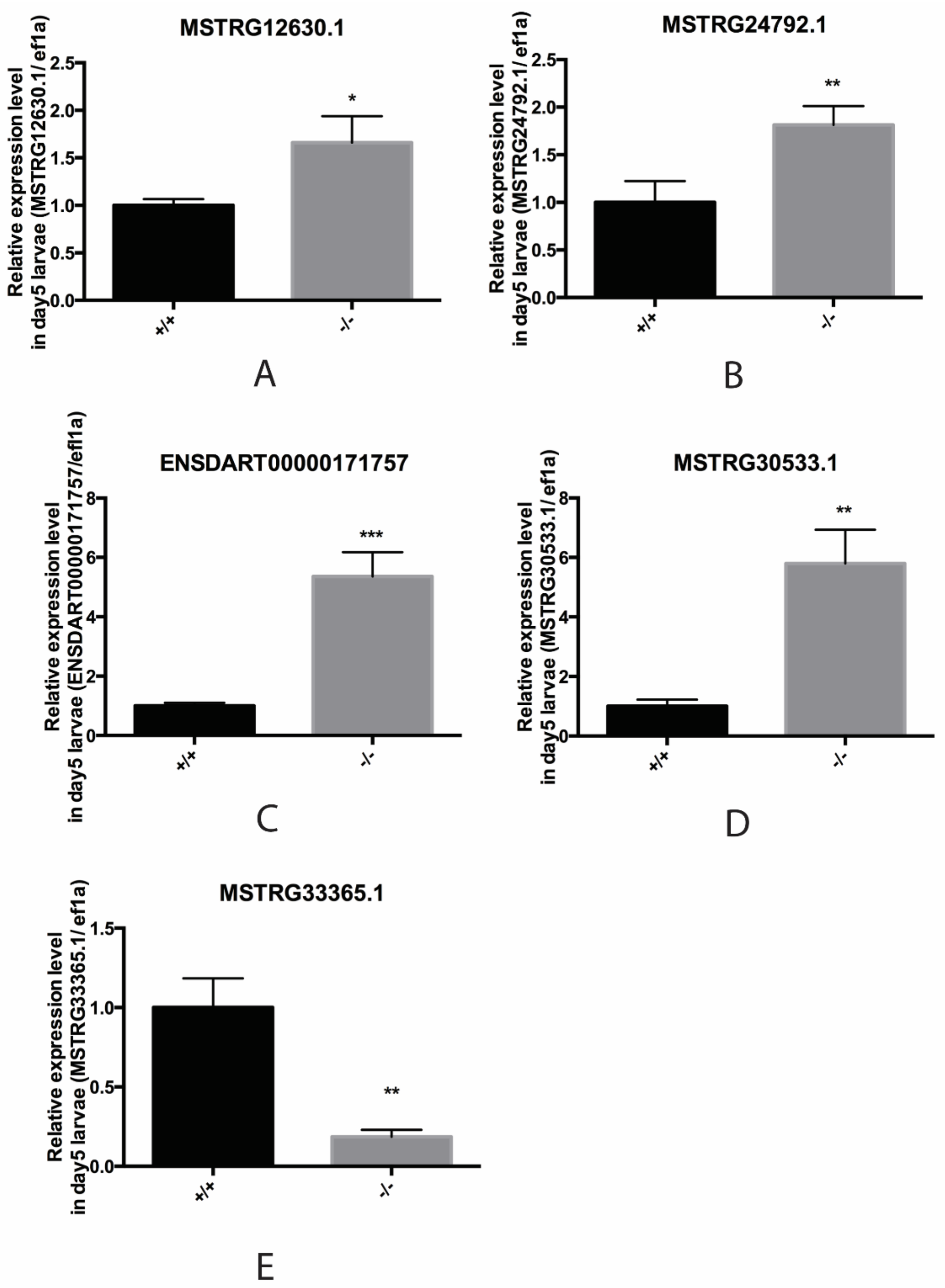

Figure S11. Immunoblotting study for validation of the correlation between Ybx1 expression and ENSDART00000171757 lncRNA knockdown. A: western blotting results of Ybx1 between cMO and ENSDART00000171757_MO (20ng) injected day5 larvae samples, larvae homogenized were with normal morphology, repeated three times. Beta-Actin expression level was used as reference. B: Relative intensity of Ybx1/Actin in day5 ENSDART00000171757_MO injected larvae, cMO injected larvae were as the control. **: p value< 0.01. Error bars indicate SD. Wilcox test used to determine significance or lack thereof.

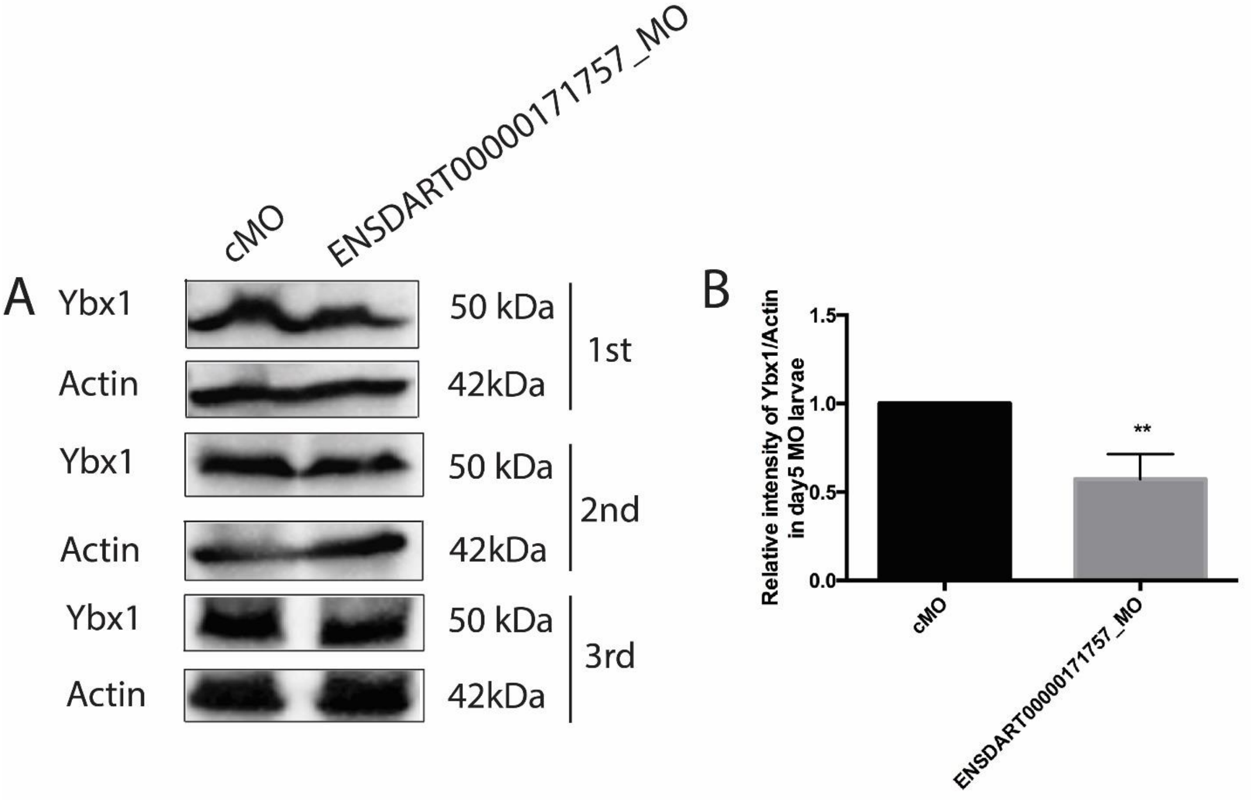

Supplement: Supplementary file 1 — Table S1. Basic statistics of zebrafish RNA‐seq data before and after quality trimming. Table S2. Basic statistics of assembly results of transcriptome in zebrafish. Table S3. Basic statistics of quality assessment of assembled transcripts achieved by DETONATE. Table S4. Potential interacted proteins of lncRNA ENSDART00000171757 predicted from catRAPID server. Fig. S1. Heatmap of DE transcripts detected in comparison between day5_ybx1+/+ and day5_ybx1−/−. Fig. S2. Volcano plot of DE transcripts detected in comparison between day5_ybx1+/+ and day5_ybx1−/−. Fig. S3. Heatmap of DE transcripts detected in comparison between day5_ybx1+/+ and day6_ybx1+/+. Fig. S4. Volcano plot of differentially expressed transcripts detected in comparison between day5_ybx1+/+ and day6_ybx1+/+. Fig. S5. Sample clustering to detect outliers. Fig. S6. Analysis of network topology for various soft‐thresholding powers. Fig. S7. Clustering dendrograms of transcripts, with dissimilarity based on topological overlap, together with assigned module colors. Fig. S8. Visualizing the gene network using a heatmap plot. Fig. S9. Scatterplots of Gene Significance (GS) for recurrence versus Module Membership (MM) in the yellow module (A) and black module (B). Fig. S10. Reverse transcription and real‐time quantitative PCR to validation of DE lncRNA identified by RNA‐seq between day 5 ybx1 +/+ and ybx1 −/− larvae. Fig. S11. Immunoblotting study for validation of the correlation between Ybx1 expression and ENSDART00000171757 lncRNA knockdown. [file FEB4-11-1259-s001.pdf]
